# Tailorable Curie Temperature in Zinc Ferrite Nanoparticles With Finely Tunable Induction Heating Profiles Between Room Temperature and 250°C

**DOI:** 10.1002/smll.202513314

**Published:** 2026-02-23

**Authors:** Leoni Luthardt, Stephan Müssig, Katrin Hurle, Andreas Zink, Xin Zhou, Benjamin Apeleo Zubiri, Erdmann Spiecker, Karl Mandel

**Affiliations:** ^1^ Chair ‘Particle‐Based Materials Chemistry’ Section Materials Chemistry Department of Chemistry and Pharmacy Friedrich‐Alexander‐Universität Erlangen‐Nürnberg (FAU) Egerlandstraße 1 Erlangen Germany; ^2^ GeoZentrum Nordbayern, Mineralogy Friedrich‐Alexander‐Universität Erlangen‐Nürnberg (FAU) Schlossgarten 5a Erlangen Germany; ^3^ Institute of Micro‐ and Nanostructure Research (IMN) & Center for Nanoanalysis and Electron Microscopy (CENEM) Friedrich‐Alexander‐Universität (FAU) Erlangen‐Nürnberg IZNF Cauerstraße 3 Erlangen Germany

**Keywords:** Curie temperature, induction heating, iron oxide nanoparticles, spray‐drying, tunable maximum heating temperature, zinc doping, zinc ferrites

## Abstract

Magnetic nanoparticles (NPs) are widely studied as heat mediators in induction heating in fields like hyperthermia, drug delivery, debonding‐on‐demand, and industrial processing, where precise thermal control is essential. By adjusting NP size, morphology, and composition, magnetic characteristics including the Curie temperature are engineered to define heating thresholds and prevent overheating. However, achieving customizable induction heating behavior, particularly below 100°C, remains challenging. Herein, a scalable synthesis of nontoxic zinc (Zn) ferrite NPs (Zn*_x_* Fe_3−_ *_x_* O_4_) with freely tailorable induction heating temperatures between room temperature and 250°C is presented. The heating performance is governed by two key parameters: Zn doping level and postsynthesis annealing temperature, with remarkably high Zn contents up to *X* = 0.75. Higher annealing temperatures and lower Zn contents yield higher maximum heating temperatures. These trends are observed both in dried NPs and dispersions, with the latter combining exceptional colloidal stability and effective heating performance. Furthermore, the heating temperature can be adjusted externally by varying the amplitude of the applied alternating magnetic field, providing further thermal control. This study establishes a versatile strategy for designing zinc ferrite NPs with precisely adjustable induction heating across a broad temperature range, enabling applications from high‐temperature industrial processes to low‐temperature biomedical use.

## Graphical abstract

A spray‐drying‐based synthesis enables the formation of zinc ferrite nanoparticles with customizable induction heating from room temperature to 250°C by varying the zinc content and postsynthesis annealing temperatures. The aqueous dispersions are colloidally stable and heat up to biologically relevant thresholds. Hence, this approach offers a scalable and versatile method for controlled thermal applications from biomedicine to high‐temperature processing.

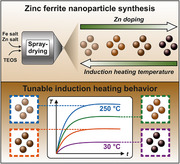

## 1 Introduction

Magnetic nanoparticles (NPs) are widely employed as heat mediators due to their ability to produce heat once exposed to an alternating magnetic field (AMF), i.e., induction heating. Heat generation results from the reorientation of the NPs themselves or their magnetic moments in response to the AMF, as well as frictional losses during this process [1]. This ability is universally exploited in use cases such as magnetic hyperthermia [2, 3, 4] and drug delivery [5, 6, 7]. Beyond biomedical use, the fast, contactless, and energy‐efficient character of induction heating [8] has recently driven interest toward broader fields including debonding on demand [9, 10, 11], catalysis [12, 13, 14, 15], selective heating in materials processing [16, 17, 18], or inductive curing [19, 20, 21].

For all these applications, tuning the properties of the employed NP species is crucial to actively control the maximum reached temperatures during induction heating, i.e., the Curie temperature (*T*_C_). The *T*_C_ is typically defined as the temperature at which a ferri‐ or ferromagnetic material loses its magnetic order and becomes paramagnetic [22]. In practice, when magnetic NPs reach their *T*_C_, their heating temperature remains the same at a thermal plateau, thus enabling self‐regulated induction heating, as the NPs “switch off” when reaching the *T*_C_, cool down minimally, and then proceed to “switch on” again, repeatedly [23, 24].

This aspect becomes particularly significant when considering that, in conventional induction‐heating systems, the temperature ultimately attained is governed by a multitude of interacting parameters, including the applied power, the distance to the inductor, the rate of heat dissipation, the surrounding medium, and many more [25, 26]. Consequently, accurate prediction and control of the final temperature are typically difficult. In contrast, *T*_C_‐regulated systems exhibit a fundamentally different behavior: even under conditions of maximal power input and optimal coupling, the temperature cannot exceed an intrinsic upper limit defined by the *T*_C_. The presence of this inherent, physically governed limit not only ensures reliable self‐regulation but also provides a critical safety advantage by preventing overheating—a feature that clearly distinguishes these materials from systems lacking such a self‐restricting mechanism. Seen from a different perspective, if it is necessary to make sure to set a certain temperature, a system with a fitting *T*_C_ could be chosen and a power input selected that ensures that the system is driven into the regime of maximum heating. Therefore, the *T*_C_ can act as a material‐inherent safety switch, as long as the order of the magnetic phase transition and the specific magnetization‐temperature profile is accounted for.

For example, biological applications typically require mild heating conditions in the range between 40 and 50°C for magnetic hyperthermia [28, 29, 30]. By maintaining these, cytotoxic effects are produced for tumor cells, while simultaneously preventing collateral damage, as uncontrolled overheating would result in protein denaturation and thermally caused injuries to the surrounding healthy tissue [31, 32]. Contrarily, technical and industrial applications like debonding on demand or catalysis often depend upon higher working temperatures, typically even beyond 200°C [15, 33, 34, 35]. Nonetheless, temperature control remains crucial, so as not to thermally damage sensitive polymer matrices during debonding or cause sintering, increase the amount of side fractions, or deactivate catalytically active substrates in catalysis [36, 37].

Conventionally, NP properties are tailored during their synthesis to achieve a finely tunable *T*_C_ by influencing morphology [38], size [39, 40], doping elements [41, 42], or interparticle interactions [43, 44]. As an alternative, postsynthesis treatment such as annealing [42], surface modification [18], or pH adjustment [45] can also affect NP characteristics. A suitable synthesis method that combines various levels of freedom in tuning the NP properties is spray‐drying, a kinetically controlled process during which precursors of choice are atomized by a nozzle into a hot chamber where the dispersing liquid is evaporated [46]. This procedure leads to the forced assembly of the droplet contents and therefore allows the combination of species independent of their chemical preference. Not only does it provide an easily scalable set‐up and reproduction of the synthesis procedure, but also ensures homogeneous mixing of inexpensive precursors and allows versatility in postsynthesis treatments including homogeneous annealing. In this regard, considering NP synthesis, high doping levels of foreign atoms can be integrated into lattices, which is difficult to achieve with other methods [47, 48, 49].

Hence, spray‐drying was already utilized in a former publication from our group for the synthesis of cobalt (Co‐) and mixed Zn‐ and Co‐doped ferrite NPs [42]. Depending on the dopants and thermal treatment, maximum induction heating temperatures could freely be tuned between 150 and 350°C. This approach presented an unprecedented degree of control over induction heating temperatures due to the flexibility given by the synthesis itself as well as postsynthetic steps.

Additionally, other approaches have been established in literature that display tailoring the *T*_C_ between 40 and 50°C for manganese perovskite [50] and Mg_1+_ *_x_* Fe_2−2_ *_x_* Ti*_x_* O_4_ ferrite NPs [51], between 100 and 400°C for Zn‐substituted manganese ferrite systems [18, 52], and between 600 and 900°C in cobalt–nickel (CoNi) NPs [12]. While all these systems display tunable induction heating behavior, major downsides persist. On the one hand, it remains a challenge to address both low and high temperature limits, meaning both below and above 100°C, with the same system, as many NPs display insufficient thermal stability, especially also for higher temperature regions. On the other hand, oftentimes, chosen synthesis routes are limited by availability or expensive set‐ups, whereas others rely on employing Co in magnetic NPs that has limited availability [53] and can be harmful both the environment and the human body [54, 55, 56, 57, 58, 59].

Consequently, the overall goal is to find a scalable and reproducible route to synthesize magnetic NPs that are tunable in terms of their *T*_C_ for low and high thermal thresholds alike. Simultaneously, it should employ only nontoxic elements as dopants like Zn that is further characterized by biocompatibility [60, 61], availability [62], and lower cost [63]. Albeit being highly promising candidates, interestingly, Zn ferrites are less studied in this context. The targeted customization of Curie temperatures for this NP species over a broader temperature range has been shown in particular for one example, where corresponding NPs were synthesized via a coprecipitation method incorporating different amounts of Zn^2+^ [64]. Yet, the tailored Curie temperatures are relatively separated, impeding fine‐tuning, and have not been tested in induction heating. Altogether, Zn ferrite NPs remain underexplored for tunable induction heating behavior, despite the promising prospective of lower temperature levels.

Consequently, in this work, we adapted a spray‐drying‐based synthesis originally utilized for ε‐Fe_2_O_3_ [65] and modified for Co/ZnCo ferrite NPs [42] to synthesize Zn ferrite NPs (Zn*_x_* Fe_3−_ *_x_* O_4_) by only employing Zn and iron (Fe) salt precursors in different ratios, together with tetraethoxysilane (TEOS) relevant for matrix formation in the process. Remarkably, employing this synthesis route for Zn ferrite NPs allowed the incorporation of unusually high Zn doping levels of up to *X* = 0.75. For each ratio, NP formation was controlled postsynthesis by varying the annealing temperatures, yielding adjustable maximum induction heating thresholds between room temperature and 250°C. Furthermore, the resulting species are suitable for dispersion‐based induction heating due to their exceptional colloidal stability and good specific absorption rate (SAR) values in aqueous medium. Generally, we showcase that Zn ferrite NP properties are precisely tailored on multiple hierarchy levels, rendering these NPs promising candidates for controlled induction heating in both low and high thermal regions.

## 2 Results and Discussion

### 2.1 Synthesis of Differently Doped Zinc Ferrite Nanoparticles

**FIGURE 1:**
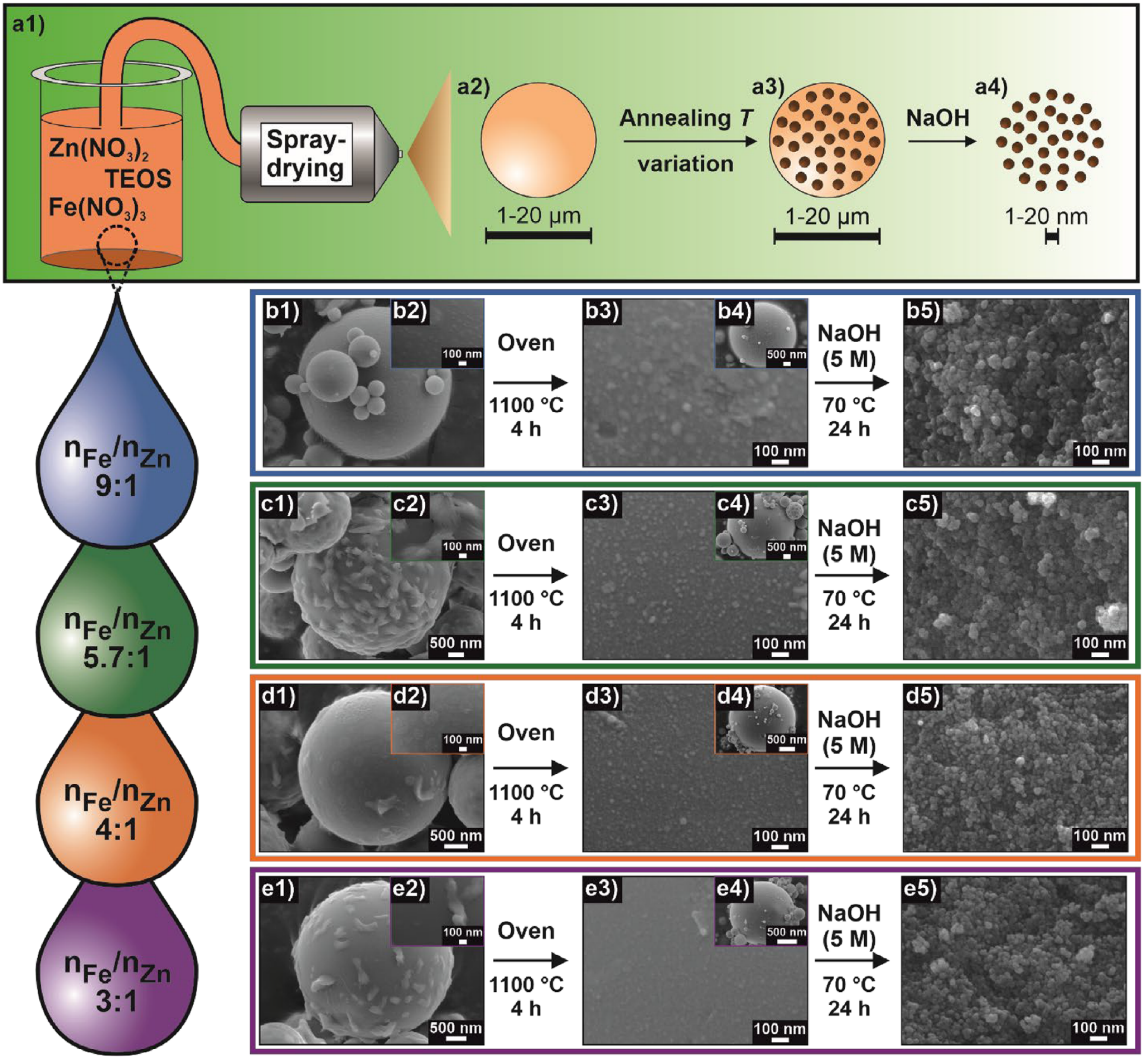
Spray‐drying‐based synthesis of Zn ferrite NPs. (a) Schematic description of the synthesis process, during which precursors are spray‐dried (a1) to yield microparticles (a2) that are further annealed. Doing so, magnetic species are formed inside the SiO_2_ matrix (a3) that are isolated by caustic treatment (a4). Varying molar ratios of Fe to Zn precursors have been tested, including 9:1 (b), 5.7:1 (c), 4:1 (d), and 3:1 (e). For all ratios, spray‐drying yields microparticles (b1–e1) with a surface that appears smooth on a nanoscale level (b2–e2). After oven treatment, magnetic species are formed in the matrix, as seen on the surface (b3–e3), however, the overall spherical microparticle shape remains (b4–e4). After treatment with NaOH, individual NP species are isolated in each case (b5–e5).

The synthesis of the presented Zn ferrite NPs in this work is modified from a spray‐drying‐based synthesis of ε‐Fe_2_O_3_ first introduced by Jo et al. [65]. Herein, corresponding precursors, in this case Zn(NO_3_)_2_, Fe(NO_3_)_3_, and TEOS, are spray‐dried from a water/ethanol mixture (Figure 1a1) to yield microparticles (Figure 1a2) that are further annealed at varying high temperatures, which lead to the emergence of magnetic species inside the formed silica (SiO_2_) matrix (Figure 1a3). As a final step, the SiO_2_ matrix is dissolved in a caustic environment to isolate the desired NP species and to be able to ultimately obtain them as a colloidally stable nanodispersion (Figure 1a4).

In the approach presented here, the molar ratio of Fe to Zn was varied in four stages gradually increasing the amount of Zn, namely, 9:1, 5.7:1, 4:1, and 3:1. In all cases, the molar ratio of the Fe and Zn precursors to TEOS was maintained at 0.4 to guarantee a constant and sufficiently large amount of matrix for NP formation. All ratios were processed by using the same parameters during spray‐drying, where the feed mixture was atomized into the hot chamber (∼100°C) to precipitate its contents into spherical, SiO_2_‐based microparticles (Figure 1b1–e1). Across all dopings, the microparticles presented a smooth surface, even though for some ratios, salt crystals from the precipitated precursors were still visible (Figure 1b2–e2).

Subsequently, the resulting species were annealed at varying temperatures between 1000 and 1100°C in a furnace for 4 h each. While for each ratio, the annealing process was performed at six different individual temperatures in this thermal range, the synthesis steps in Figure 1 are exemplarily shown only for the highest employed temperature, i.e., 1100°C. This temperature treatment led to the formation of NP species at each ratio, as seen on the microparticle surface (Figure 1b3–e3), whereas the overall spherical morphology of the microparticle matrix remained intact (Figure 1b4–e4). Additionally, no salt crystals were visible on the surface anymore after the annealing process. The formation of ferrite NP species was confirmed by the emergence of a nonzero magnetic moment after the oven treatment, while none was measurable in the pristine state directly after spray‐drying (Figure S1). For ratios of 9:1 and 5.7:1, a magnetic moment of around 20 emu g^−1^ was detected, for higher Zn contents in 4:1 and 3:1 ratios, the magnetic moment was diminished to 10 emu g^−1^.

The respective NP species were each isolated by treatment of the annealed microparticles with NaOH (5 m) at 70°C for 24 h, followed by purification steps. As the nonmagnetic SiO_2_ matrix is dissolved in the process, the magnetic moment is even further increased after caustic treatment, revealing values of 45–55 emu g^−1^ for ratios of 9:1 and 5.7:1, respectively, and values of 30–40 emu g^−1^ for 4:1 and 3:1 ratios with a higher Zn share (see Figure S1). Scanning electron microscopy‐energy‐dispersive X‐ray (SEM‐EDX) analysis reveals that Zn is present in every sample independent of the doping amount, as evidenced by the Zn Kα peak in the EDX spectrum (Figure S2a) and corresponding qualitative count maps (Figure S2b). With increasing Zn doping, the Zn Kα peak increases in intensity, indicating a higher amount of Zn built into the structure of the ferrite NPs, which is simultaneously confirmed by an increasing Zn/Fe ratio according to Zn Kα and Fe Kα peak intensities (see Table S1).

To estimate the final Fe to Zn in the individual NP species, inductively coupled plasma optical emission spectrometry (ICP‐OES) measurements were performed, while a magnetite crystal structure is assumed based on previous ferrite species synthesized by spray‐drying [42]. In accordance with the trend observed in EDX, Zn shares increased and Fe shares decreased with a decreasing Fe:Zn molar ratio during the synthesis procedure (Table S2). Taking deviations due to synthetical processes into account, the final sum formulas for the Zn‐doped magnetite structure of each doping level are a good approach for the calculated theoretical values in each case and are further referred to as follows: Zn_0.3_Fe_2.7_O_4_ for a 9:1 Fe:Zn molar ratio (abbreviated as Zn0.3 NPs), Zn_0.45_Fe_2.55_O_4_ for a 5.7:1 ratio (abbreviated as Zn0.45 NPs), Zn_0.6_Fe_2.4_O_4_ for a 4:1 ratio (abbreviated as Zn0.6 NPs), and Zn_0.75_Fe_2.25_O_4_ for an employed 3:1 ratio (abbreviated as Zn0.75 NPs).

### 2.2 Mechanism of the Zinc Ferrite Nanoparticle Formation During High‐Temperature Annealing

**FIGURE 2:**
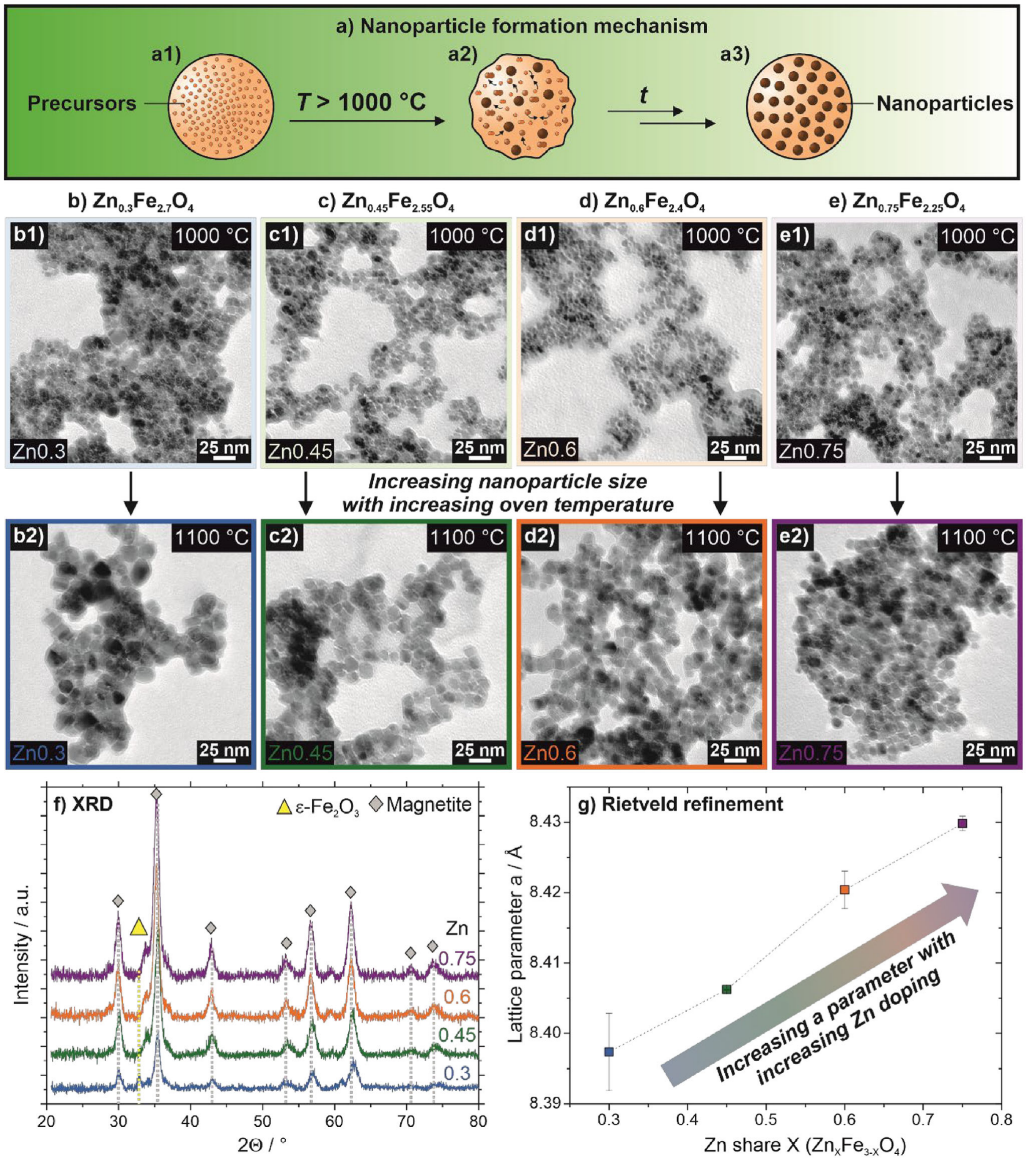
Mechanism of the Zn ferrite NP formation. (a) Nitrate precursors are precipitated to oxides in the newly formed SiO_2_ matrix (a1), that react to Zn ferrites and coalesce once the glass transition temperature of SiO_2_ (1000°C) is exceeded (a2), forming the desired NP species during the annealing time (a3). (b–e) TEM images reveal an increase in particle size when comparing NPs annealed at 1000 (b1–e1) and 1100°C (b2–e2) for each doping, as seen for Zn0.3 (b), Zn0.45 (c), Zn0.6 (d), and Zn0.75 NPs (e). (f) X‐ray diffractograms display a magnetite structure for every doping with minor contents of ε‐Fe_2_O_3_ for lower Zn contents. (g) Rietveld refinement reveals an increasing *a* parameter of magnetite with an increasing Zn share incorporated into the NPs.

The mechanism of NP formation in the spray‐drying‐based synthesis with subsequent annealing steps has been thoroughly discussed in our previous publication [42]. It is expected that the underlying particle formation procedure here is of similar nature, even though no Co is present as opposed to our former work. In the first step during spray‐drying, a porous SiO_2_ matrix is formed from the TEOS precursor. Corresponding nitrate precursors are decomposed to oxides, inducing the formation of γ‐Fe_2_O_3_ and ZnO that react in the matrix cavities to Zn ferrite NPs (Figure 2a1) [66, 67]. However, this effect is neglectable prior to high temperature treatment, as SiO_2_ acts as a spacer for the newly formed species and prevents their movement and consequently aggregation in the rigid matrix. Only above the glass transition temperature of SiO_2_ (∼1000°C), the matrix becomes soft, and the provided thermal energy allows for diffusion of NPs and their coalescence to larger species (Figure 2a2), inducing magnetic NP formation inside the matrix over the entire time frame of the annealing process (Figure 2a3) [68, 69, 70, 71]. Furthermore, it has been found elsewhere that Zn ferrites are stable within a SiO_2_ matrix at temperatures up to 1100°C [71, 72].

With increasing annealing temperature, the crystallite size of ferrites is known to enhance [70, 71, 72], presumably due to the minimization of the overall energy of the system by the induced coalescence to larger NPs [73]. This is in accordance with transmission electron microscopy (TEM) images recorded for all doping levels that display an NP growth in the observed temperature range, starting at 7–8 nm when annealed at 1000°C and increasing to 12–15 nm at 1100°C (Figure 2b–e; see Figure S3 for TEM images of all dopings annealed at temperatures between 1020 and 1060°C, and Figure S4 for particle size distributions of all dopings at all annealing temperatures). Additionally, taking minor deviations for the lowest doped Zn0.3 NP sample into account, crystallite sizes as determined by Rietveld refinement show the same trend (see Table S3).

Furthermore, we propose that the temperature‐enhanced particle growth takes place as the matrix becomes viscous on a faster time scale when exposed to higher temperatures. This allows more NPs to sinter together quicker, creating larger NPs in the same time scale, whereas with lower temperatures, the growth is more restricted by SiO_2_ confinement. It is to be noted that in each case, the overall NP size distribution is quite broad due to the kinetically controlled particle formation process. Moreover, the presented NPs display a quasispherical morphology at all annealing temperatures for all Fe/Zn ratios, as it is typical for Zn ferrite species [74, 75, 76], with polyhedral deviations known for high‐temperature synthesis routes [77, 78].

X‐ray diffraction (XRD) analysis and Rietveld refinement of dried NPs of all doping levels reveal a crystal pattern fitting to a magnetite/maghemite crystal structure with characteristic reflexes at 30.0°, 35.4°, 53.3°, 56.7°, 62.2°, 70.7°, and 73.9° 2Θ (Figure 2f) [79]. Since the diffraction patterns of maghemite and magnetite are quite similar, these two phases are difficult to distinguish by XRD. However, maghemite has two additional reflexes at around 23.5° and 26° 2Θ, which are not visible in the diffraction patterns of the investigated samples. Hence, it is assumed that at least the major fraction is actually magnetite, and the refinement was thus performed with the magnetite structure. For the lowest doped sample, contributions of ε‐Fe_2_O_3_ that make up about 33 wt% are measurable at 32.8°, while a side fraction of hematite is apparent for Zn0.45 NPs (see Rietveld refinement in Table S4) [80]. The comparatively high share of ε‐Fe_2_O_3_ in the NPs doped with the lowest amount of Zn is presumably due to the originally modified spray‐drying‐based ε‐Fe_2_O_3_ synthesis. We hypothesize that a certain minimum amount of Zn doping is crucial to achieve a pure magnetite structure without ε‐Fe_2_O_3_ share, as seen for the higher doped samples.

These findings are in correspondence with literature‐known Zn ferrites typically demonstrating a normal spinel structure with Zn^2+^ preferentially on the tetrahedral (A) and Fe^3+^ on the octahedral (B) sites. The absence of reflexes at 34.4°, 36.3°, 47.6°, and 68.0° for all dopings further confirms that Zn^2+^ ions were successfully incorporated into the lattice structure and no (measurable) ZnO species is present [64]. This composition is maintained to large extents independent of the Zn doping level, while it has been reported elsewhere that the transformation to a normal spinel structure is increased with higher Zn dopings [81]. When considering XRD analysis recorded for different annealing temperatures between 1000 and 1100°C at the same doping level, as exemplarily done for Zn0.3, it becomes apparent that the magnetite/maghemite structure with a ε‐Fe_2_O_3_ share is sustained across all thermal treatments (Figure S5). However, reflexes gain in intensity with higher annealing temperatures, indicating a higher level of crystallinity which is a known phenomenon, as lattice strain and defects are reduced [67, 82, 83].

Rietveld XRD refinement data further reveals that with increasing Zn share within the synthesized NPs, the *a* parameter of magnetite is increasing from 8.3974 to 8.4298 Å (Figure 2g, for exact values see Table S5), which is in accordance with literature reports for Zn‐substituted magnetites [84, 85]. This is due to the larger ionic radius of Zn^2+^ compared to Fe^3+^ on the tetrahedral sites, leading to an overall expansion of the lattice and further confirming the incorporation of Zn into the NP structure.

**FIGURE 3:**
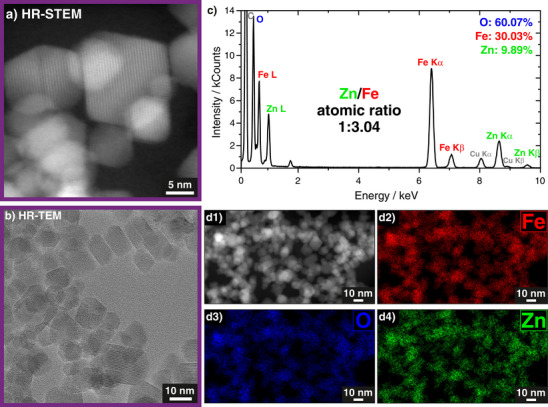
Structural investigation of Zn_0.75_Fe_2.25_O_4_ NPs by HAADF‐STEM, same for Figure S6 (a) and HR‐TEM (b), displaying a spinel lattice with no indication for secondary or amorphous phases. STEM‐EDX (c,d) showcases an even spatial distribution of Zn throughout the NPs, with a calculated Zn:Fe atomic ratio of 1:3.04, which matches the nominal stoichiometry very well. The X‐ray peaks at ∼8 keV, ∼8.9 keV are related to Cu Kα, and Cu Kβ, mainly contributed by the Cu TEM grid. The X‐ray peak at ∼1.7 keV is related to Si, mainly contributed from the detector. The huge peak at ∼0.3 keV is attributed to C.

It is notable that, using the newly established spray‐drying‐based synthesis route for Zn ferrite NPs, remarkably high Zn substitution levels (up to *X* = 0.75) can be incorporated into the magnetite spinel structure. This is confirmed by high‐resolution TEM (HR‐TEM) and high‐angle annular dark‐field scanning TEM (HAADF‐STEM) measurements, as well as STEM‐EDX, as displayed in Figure 3 exemplarily for the highest doped sample, i.e., Zn_0.75_Fe_2.25_O_4_. HAADF‐STEM and HR‐TEM (Figure 3a,b) support that no amorphous or secondary phases are formed next to the magnetite/maghemite phase, while STEM‐EDX (Figure 3c,d) displays an even spatial distribution of Zn throughout the NPs. The calculated Zn:Fe atomic ratio from this data matches very well with the nominal stoichiometry, therefore verifying the successful incorporation of the comparatively high Zn shares into the spinel lattice. The high crystallinity and uniform Zn spatial distribution for the NPs with lower Zn doping concentrations were likewise confirmed by TEM investigation on the Zn_0.75_Fe_2.25_O_4_ nanoparticles, as shown in Figure S6.

This is further confirmed by the Rietveld‐refined XRD data displaying no apparent side fractions, together with an almost linear increase in the *a* parameter with increasing Zn doping levels, that has, in addition to the Rietveld data, also been confirmed with selected area electron diffraction (SAED). The obtained data is showcased in Table S6 (see also Figure S7 for raw SAED patterns). A potential reason for no appearance of secondary phases is the high‐temperature synthesis procedure, in which formed ZnO reacts further with iron oxide to form zinc ferrites, as explained above. Such high Zn dopings as presented herein exceed the typical substitution limit (*X* ∼ 0.3–0.5) reported in literature for Zn*_x_* Fe_3−_ *_x_* O_4_ species [86, 87], indicating that our synthesis conditions enable extended Zn incorporation into the lattice, which is a remarkable feature of using spray‐drying as a synthesis route.

### 2.3 Induction Heating Behavior of Dried Zinc Ferrite Nanoparticles

Independent of the doping level, NP formation occurs during the annealing step after spray‐drying. In this regard, it has been found previously for Co and ZnCo ferrites that magnetic characteristics and therefore the maximum reached induction heating temperature can be tuned between 200 and 350°C or 150 and 220°C, respectively, by varying the oven temperature during the thermal treatment [42].

**FIGURE 4:**
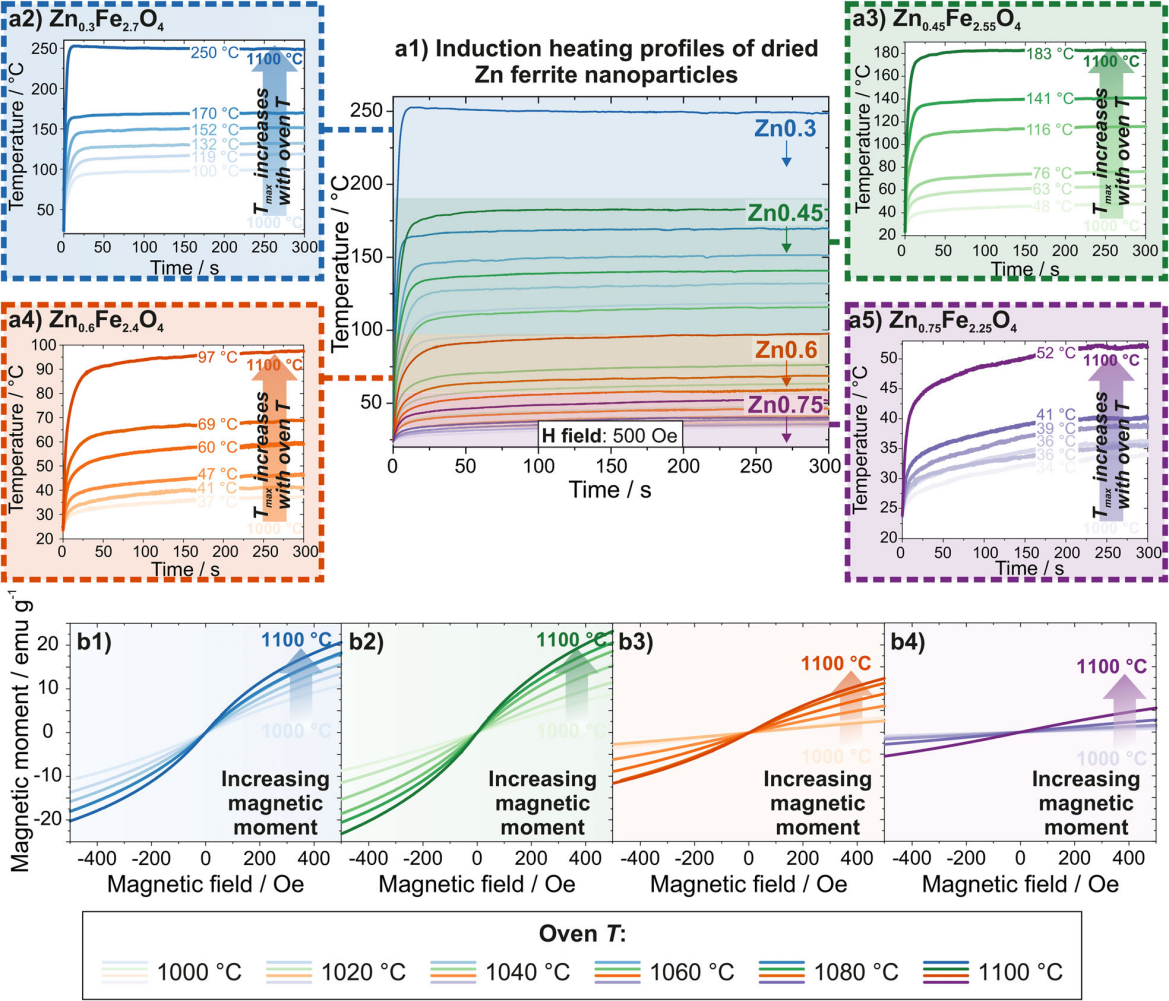
(a) Induction heating behavior and magnetic characteristics of dried differently doped Zn ferrite NPs. (a1) Induction heating curves of all species at all tested annealing temperatures displayed together, underlining that induction heating temperatures can be tailored between room temperature and 250°C. For all dopings, the oven annealing temperature was varied between 1000 and 1100°C after spray‐drying, resulting in different induction heating temperatures when subjected to an AMF afterward. Overall, the heating temperature is increased for increasing oven annealing temperature and lower Zn doping. Individual heating curves of the corresponding doping levels at all annealing temperatures are displayed for Zn0.3 (a2), Zn0.45 (a3), Zn0.6 (a4), and Zn0.75 (a5) NPs. All induction heating curves are obtained at an AMF amplitude of 500 Oe and a frequency of 1.4 MHz over a time scale of 300 s. (b) Magnetization curves obtained via VSM for Zn0.3 (b1), Zn0.45 (b2), Zn0.6 (b3), and Zn0.75 (b4) NPs at different annealing temperatures. The magnetic moment is increased with increasing oven temperature for every doping, while the overall magnetic moment decreases with dopings exceeding Zn0.45. Magnetization values are given in emu per gram of the measured powder sample mass.

For each of the four investigated Zn doping amounts herein, the oven annealing temperature was varied in 20°C steps between 1000 and 1100°C to form the investigated NPs. Depending on the utilized oven temperature, the maximum induction heating temperature of each of these NP species could be tailored when subjected to an AMF after annealing. The trend of a higher induction heating temperature with higher oven annealing temperatures was seen independent of the doping amount, however, variation in different temperature ranges could be achieved dependent on the doping level (Figure 4a1). Consequently, the highest possible maximum induction heating temperatures were attained for the lowest Zn doping (Zn0.3), variable between 100 and 250°C (Figure 4a2) while the highest doping (Zn0.75) resulted in the lowest induction heating temperatures of 30 to 50°C (Figure 4a5). Induction heating temperatures for Zn0.45 NPs could be tuned between 50 and 180°C (Figure 4a3), and those for Zn0.6 NPs between 35 and 100°C (Figure 4a4).

The obtained maximum induction heating temperatures (for each doping and each oven annealing temperature, respectively) are in accordance with Curie temperatures measured via static magnet‐supported thermogravimetric analysis (TGA) that revealed the trend of an increasing Curie temperature with increasing oven temperatures for every doping (Figure S8). Simultaneously, comparing different doping levels, the overall Curie temperature is reduced with increasing Zn share, in agreement with recorded induction heating curves. Literature confirms that Curie temperatures decrease with increasing Zn doping [64] caused by weaker magnetic exchange interactions due to more diamagnetic Zn^2+^ ions in the crystal lattice and therefore increased lattice parameters [88, 89]. Similarly, inductive heating rates are increased with higher oven annealing temperatures and decreasing Zn doping (Table S7). For example, Zn0.3 NPs display a heating rate of 9 K s^−1^ when annealed at 1000°C, which is enhanced fourfold at an oven temperature of 1100°C to a value of 40 K s^−1^. In comparison, Zn0.75 NPs only exhibit a heating rate of 0.5 K s^−1^ at 1000°C annealing temperature that is increased to the still comparatively low value of 3 K s^−1^ when calcinated at 1100°C.

Taking all induction heating curves into account, it becomes obvious that with higher Zn dopings, the range of variability in tuning becomes smaller. However, with high Zn share, lower maximum heating temperatures are reachable that are, to the best of our knowledge, hard to address by any other means of synthesis procedures with a similar degree of flexibility. By employing Zn0.3 and Zn0.45 dopings, the temperature range between 150 and 250°C is aimed for, that is also achievable with Co and ZnCo ferrites, but omits the toxic element Co herein. An even lower thermal range between 30 and 150°C that is not attainable with solely ZnCo ferrites can be addressed by using specifically annealed Zn0.45 as well as Zn0.6 and Zn0.75 NPs. This underlines the great benefit of this synthesis approach that lies in the variable tuning of the maximum induction heating temperatures between 30 and 250°C by adapting the doping level during spray‐drying on the one hand and the oven temperature during postsynthetic annealing on the other hand. Induction heating with Zn ferrites synthesized in this manner therefore offers a high range of variability, easy adjustments, nontoxicity, and possible low induction heating temperatures that are favorable in application‐based scenarios where other parts in the surroundings of the employed NPs should not take harm by overheating.

All investigated Zn ferrite species are superparamagnetic according to vibrating sample magnetometer (VSM) measurements recorded between −500 and 500 Oe (to mimic the AMF applied during induction heating) that displayed no measurable coercivity (Figure 4b). This superparamagnetic character is in accordance with literature reports [64]. For Zn0.3 and Zn0.45 dopings, the maximum magnetic moment reached in this magnetic range is between 20 and 25 emu g^−1^, for Zn0.6 around 12.5 emu g^−1^, and for Zn0.75 at 5 emu g^−1^. In all cases, the magnetic moment increases with increasing annealing temperature during the oven treatment. Up to a specific doping amount, the saturation magnetization rises with increasing Zn doping as the A sites are occupied with Zn^2+^ ions and the corresponding A site magnetization is decreased. At higher doping levels, the magnetic moment drops again as Zn^2+^ also starts to displace Fe^3+^ on B sites, decreasing the net magnetization [64, 84, 89]. The lower induction heating temperature with increasing doping amounts is therefore connected to the lower maximum magnetic moment in the investigated magnetic field range.

The higher maximum reached induction heating temperatures of Zn0.3 compared to Zn0.45 NPs despite the slightly higher magnetic moment of the latter at 500 Oe can be explained by considering the VSM curves recorded between −30 and 30 kOe, where the actual saturation magnetization is reached (Figure S9). It becomes obvious that Zn0.3 NPs show a pinched hysteresis loop, displaying no coercivity around 0 Oe, but developing hysteresis at higher fields. This results in a higher heating capability and thus, a higher reached maximum induction heating temperature compared to Zn0.45 that displays no such pinched hysteresis. The pinched hysteresis of specifically Zn0.3 NPs is most likely due to the remaining ε‐Fe_2_O_3_ share, as the latter typically displays a large hysteresis area [90], which is also known to be reduced when substituted with other metal ions [91, 92].

**FIGURE 5:**
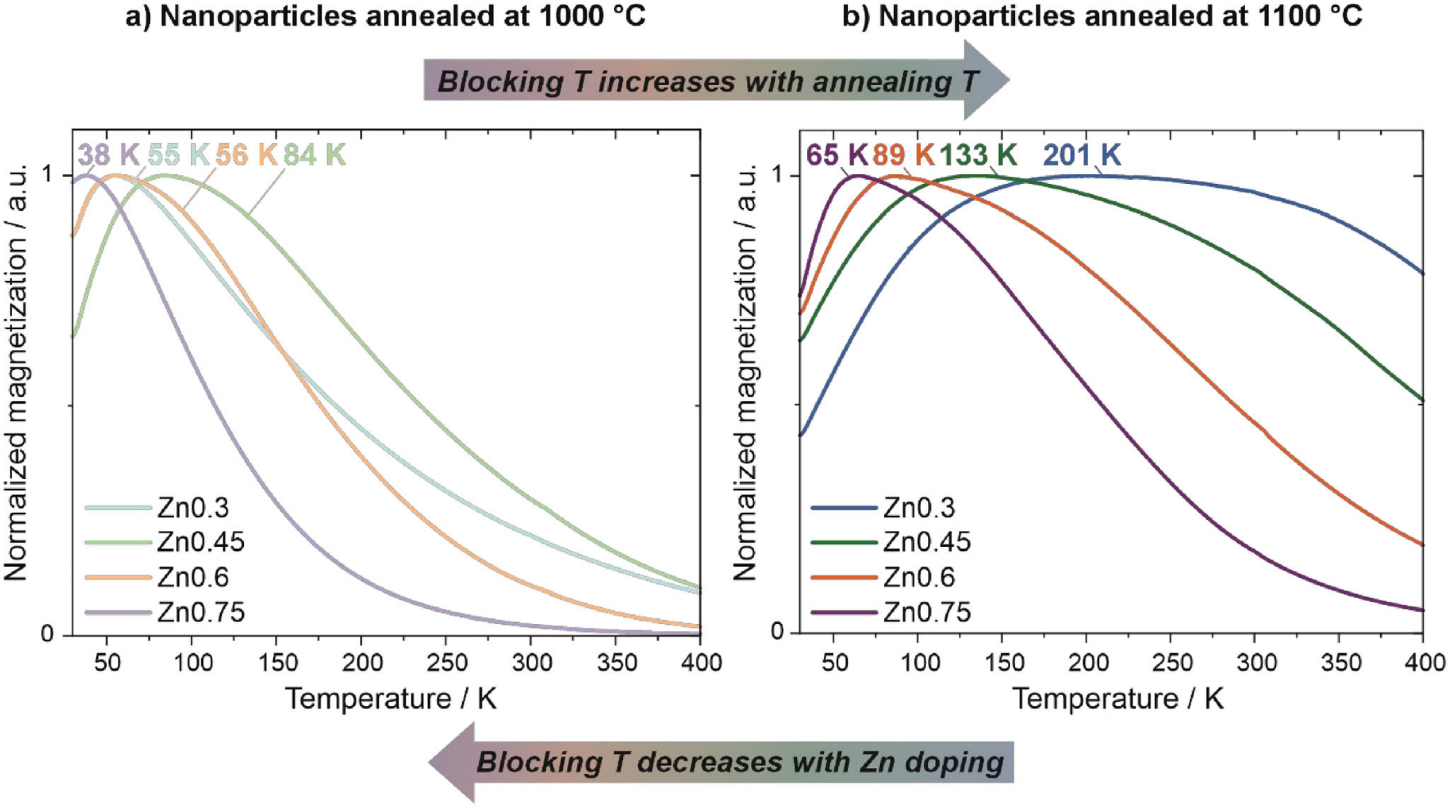
ZFC curves of Zn ferrite NPs annealed at 1000 (a) and 1100°C (b), respectively. For clarity, FC curves are not depicted and the magnetization values are normalized. The *T*_B_ of every respective sample is indicated above the corresponding curve. *T*_B_ generally increases with decreasing Zn doping and increasing oven annealing temperature. The *T*_B_ was estimated from the maximum of the ZFC curve in each case.

In addition to regular VSM measurements, ZFC/FC curves have been recorded for the samples with the lowest and highest annealing temperature for every Zn doping, respectively (Figure 5, only ZFC curves depicted for clarity). It becomes apparent that with increasing Zn share, the blocking temperature (*T*_B_) decreases (which is true independent of the annealing temperature, with Zn0.3 annealed at 1000°C being an outlier, presumably due to the ε‐Fe_2_O_3_ share). This is due to nonmagnetic Zn^2+^ replacing Fe^3+^ in the lattice, thus reducing the A–B site coupling and the overall magnetic anisotropy, which is directly proportional to *T*_B_ [93, 94]. Additionally, when comparing Figure 5a and Figure 5b, *T*_B_ increases for an increasing annealing temperature, which is in accordance with a NP diameter (*d*) increase at higher temperatures, which in turn raises the *T*_B_ due to its scaling with the particle volume (*V*) (*V* ∼ *d* ^3^) [95]. Notably, the depicted ZFC curves broaden significantly at higher annealing temperatures, which is most likely attributed to the relative polydispersity in the corresponding samples [96]. The *T*_B_ of each NP species scales linearly with its volume. Hence, when a certain polydispersity is present, smaller (low‐volume) NPs will unblock at lower temperatures, while larger (high‐volume) NPs unblock at higher temperatures. This results in the *T*_B_ not presenting itself as a sharp maximum, but rather as a broad curve, constituting a continuous range of blocking temperatures. Table S8 displays the mean *T*_B_ for all depicted samples, determined from the maximum of the ZFC curve, as well as the upper *T*_B_ limit, estimated from the convergence of the FC and ZFC curves. Correspondingly, un‐normalized ZFC/FC curves for every sample are depicted in Figure S10 (see also the Supporting Information for a more detailed discussion of the ZFC/FC curves). In general, the mean *T*_B_ and upper *T*_B_ limit is diminished for higher Zn dopings and lower annealing temperatures. Broader ZFC curves for lower and narrower curves for higher Zn dopings also correlate well with the sample polydispersity (Figure S4).

Due to the superparamagnetic character of all species, additional tailoring of the resulting induction heating temperature is possible for all NPs via modulation of the AMF amplitude, opening up another level of freedom in customizing the heating temperature (Figure S11). When applying an AMF amplitude lower than the maximum (500 Oe), different points of the magnetic moment on the magnetization curve are met, as the sample is not fully excited to saturation in the induction heating device. This results in fine‐tuned and overall lower induction heating plateaus, potentially helpful for use cases where the sample is already synthesized with the corresponding doping amount, but a lower heating temperature is desired, for example when already integrated into a working environment. It is important to maintain the same frequency when modulating the AMF amplitude to obtain the plateaus presented in Figure S11, as this is another factor the final induction heating temperature depends on. In this case, the AMF amplitude was changed at a constant frequency of 1.4 MHz. For Zn0.3 NPs annealed at 1100°C, induction heating can be fine‐tuned between 110 and 250°C, that of Zn0.45 NPs between 75 and 180°C. The higher dopings allow smaller thermal variations between the tuned temperatures, with Zn0.6 NPs tunable between 30 and 100°C, and Zn0.75 NPs between 25 and 50°C. The induction heating temperature therefore depends on three factors that are relevant on different hierarchies: the synthesis (regarding the amount of Zn doping), the postsynthesis modification (regarding the temperature during the oven annealing step), and the external regulation of the AMF (regarding the induction heater settings).

### 2.4 Induction Heating Behavior of Zinc Ferrite Nanoparticle Dispersions

**FIGURE 6:**
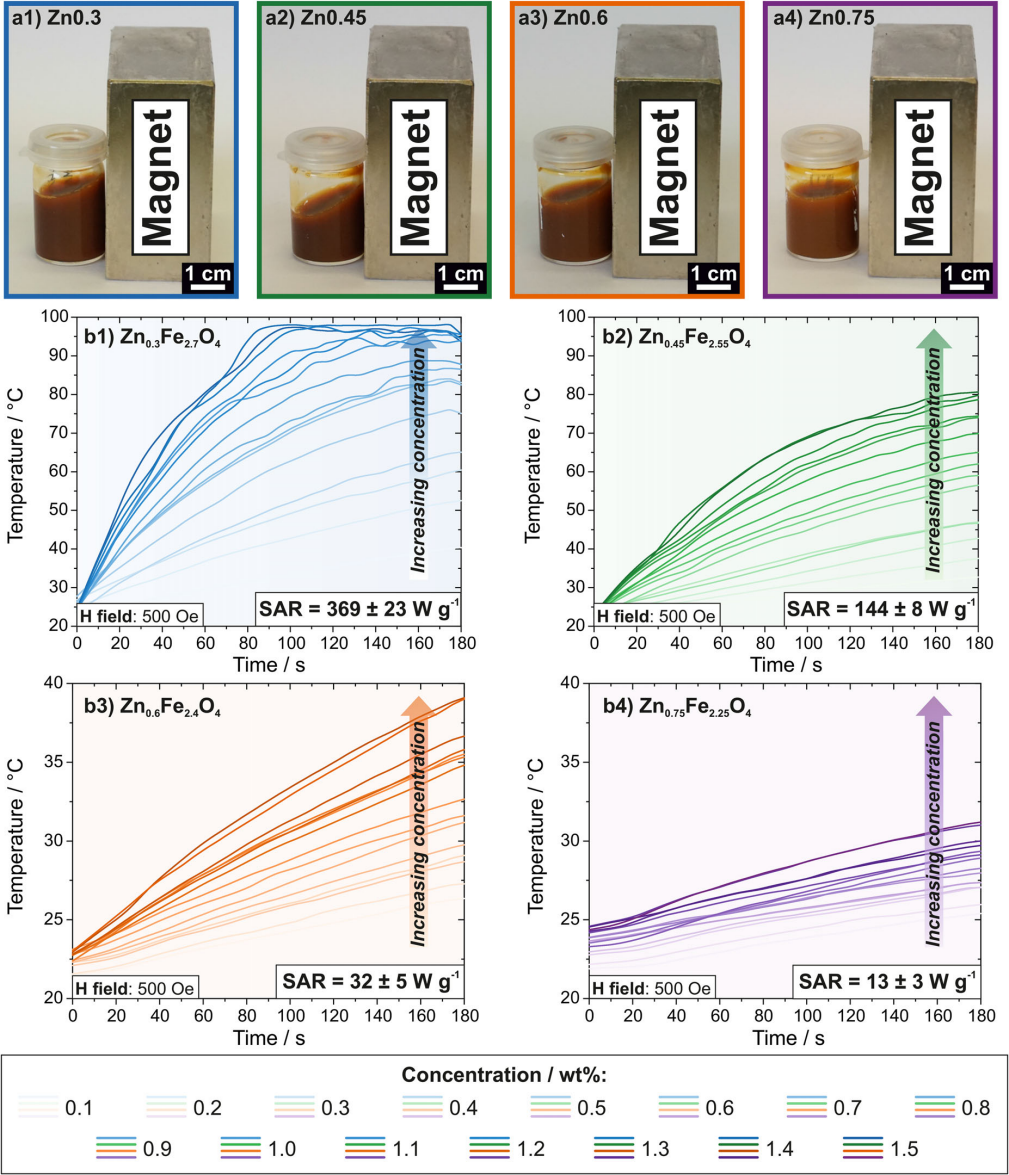
(a) Colloidal stability of Zn ferrites annealed at 1100°C in aqueous dispersion, shown for Zn0.3 (a1), Zn0.45 (a2), Zn0.6 (a3), and Zn0.75 (a4) NPs when placed next to a static magnet. (b) Induction heating in aqueous dispersion of Zn0.3 (a1), Zn0.45 (a2), Zn0.6 (a3), and Zn0.75 (a4) NPs, each annealed at 1100°C and tested for varying concentrations between 0.1 and 1.5 wt% at an AMF amplitude of 500 Oe and a frequency of 1.4 MHz. The overall reached temperature in 180 s increased for increasing concentration in every case. SAR values are given in W per gram of the utilized nanoparticle mass.

The induction heating behavior of differently doped Zn ferrite NPs has further been tested in aqueous dispersion. All dispersions independent of Zn doping are colloidally stable, which is exemplarily shown for one sample each that was annealed at 1100°C in a concentration of 1 wt% next to a magnet with a static field of approximately 4 kOe in Figure 6a. In each case, no magnetic separation of the dispersion is induced, showcasing exceptional stability that is otherwise often only found for species with low induction heating rates.

The same colloidal stability also counts for dispersions of Zn ferrites annealed at 1000°C at the same concentration, although they are significantly less attracted by a magnet due to a lower overall magnetization (Figure S12). Furthermore, the colloidal stability is confirmed by hydrodynamic particle diameters as measured by dynamic light scattering (DLS). Hydrodynamic particle sizes are comparable for each Zn doping, both annealed at 1000 and 1100°C, issuing mean values between 50 and 80 nm without formation of larger agglomerates in each case (see Figure S13 and Table S9). Larger hydrodynamic sizes compared to the actual size determined from TEM images are expected due to the remaining SiO_2_ shell, and compare well to literature‐known values for SiO_2_‐coated colloidally stable magnetic NPs [97].

When directly comparing undoped ferrimagnetic iron oxide NPs (FIONs) and, exemplarily, Zn0.3 NPs annealed at 1100°C, the stability over longer amounts of time is confirmed. While FIONs are immediately magnetically separated after even 1 s and stay separated, Zn ferrites are stable and unchanged in the presence of the magnetic field even after 3 min (Figure S14). Hence, FIONs would need another functionalization step to enhance colloidal stability, whereas it is already given after the synthesis procedure for Zn ferrites. This exceptional stability across all dopings and annealing temperatures is due to the small NP size in combination with the remaining SiO_2_ shell around the NPs that stems from the utilized synthesis route and provides electrostatic stabilization, similar as for Co and ZnCo ferrite NPs [42]. Colloidal stability allows for the measurement of the temperature during dispersion induction heating directly on the dispersion surface, as the temperature is expected to be the same in the entire dispersion, and no boiling delays are expected.

Dispersions of NPs annealed at 1100°C of all four doping levels were inductively heated in varying concentrations between 0.1 and 1.5 wt% and a total volume of 3 mL. As the samples in water became increasingly hot, steam development was visible that hampers with the infrared temperature measurement technique. Therefore, the resulting induction heating curves were smoothed with a Lowess function to make for better visibility and comparison between individual concentrations (for unsmoothed curves see Figure S15). All induction heating experiments were performed at the maximum possible field of 500 Oe over a time range of 3 min. With increasing Zn doping, a lower overall temperature was reached in dispersion heating, similar to the trend of NPs tested in their dried agglomerated state (Figure 6b). For every sample, a higher dispersion temperature was reached with increasing concentration in the same time frame. To compare the induction heating performance between samples, the SAR was approximated in W g^−1^ using the following equation:

(1) $$\mathrm{SAR}=\frac{C_{H2O}\cdot \beta }{m_{\mathrm{NPs}}}\;$$

where *m*_NPs_ is the NP mass for the respective concentration, *C*_H2O_ is the heat capacity of water as a product of *c*_H2O_ as the specific heat capacity of water (4.186 J K^−1^) and *m*_H2O_ as the mass of water at the respective NP concentration, and *β* is Δ*T*/Δ*t* for the temperature change in the initial slope during a specified time frame. The latter has been determined depending on the overall temperature change of the respective Zn dopings (Zn0.3/Zn0.45: *t* for 10°C; Zn0.6: *t* for 3°C; Zn0.75: *t* for 2°C). To determine the SAR, only values of concentrations between 1.0 and 1.5 wt% were taken into account for every doping, as it is considered to make for more reproducible and reliable results (for the SAR values across all dopings, see Table S10). A discussion of the SAR determination and the impact of AMF amplitude and frequency in correlation to the intrinsic loss power has been performed in our previous publication [42].

The best induction heating performance in dispersion, in correspondence with the results in the dried agglomerated state, was reached with Zn0.3 NPs that exhibited an SAR of 369 ± 23 W g^−1^ (Figure 6b1). While this value is lower than that of ZnCo ferrite NPs prepared by spray‐drying (2400 W g^−1^), it exceeds that of pure Co ferrite NPs (200 W g^−1^). The improved performance in aqueous dispersion compared to Co ferrites is presumably due to the superparamagnetic character of Zn ferrites and therefore no apparent coercivity in the magnetic field range of the induction heating device, which makes for more efficient heating in aqueous medium and less contribution of heat losses as opposed to Co ferrites exhibiting a significantly broader coercivity [42]. This species also shows good performance at low concentrations, as temperatures of above 50°C are reached in 3 min at concentrations as low as 0.1 wt%, whereas concentrations starting from 1.0 wt% prompt temperatures of over 70°C in a time frame as small as 1 min. In comparison, when annealed at 1000°C, Zn0.3 only display an SAR of 21 ± 4 W g^−1^ with a maximum temperature of 40°C reached in 3 min even at the highest concentration (Figure S16b). This again underlines the impact that the annealing temperature has on the induction heating performance of the resulting NPs, not only in the dried state, but also in dispersion. Furthermore, a comparison of the induction heating behavior of dispersions of all annealing temperature of Zn0.3 measured at 1.0 wt% reveals that the maximum reached temperature in 3 min increases in trend with the increasing oven temperature postsynthesis (Figure S16c).

Zn0.45 ferrite NPs also exhibit good induction heating performance in dispersion with an SAR of 144 ± 8 W g^−1^ that is comparable to that of Co ferrites (Figure 6b2). The comparatively high‐quality performance is due to their superparamagnetic character and high magnetic moment. Temperatures of 80°C are reached in 3 min at the highest concentrations of this NP species, whereas heating over 50°C is induced at still low concentrations of 0.6 wt%. When heated over a longer period of time (15 min), a temperature plateau is reached at approximately 90°C, which is almost the maximum possible temperature in water, that also further underlines the good induction heating behavior in dispersion (Figure S16a).

With Zn ferrite NPs of higher dopings (Zn0.6 and Zn0.75), significantly lower thermal thresholds are reached in 3 min with reduced SAR values of 32 and 13 W g^−1^, respectively (Figure 6b3 and Figure 6b4). For Zn0.6, temperatures up to 40°C can be achieved, while Zn0.75 ferrite NPs heat only to 32°C at the highest concentrations, whereas the lower concentrations heat barely above room temperature. Interestingly, when heated over a time scale of 15 min at 1.0 wt%, Zn0.6 ferrite NPs reach a plateau around 50°C and Zn0.75 NPs around 40°C (Figure S16xa). This shows that these species might be relevant for biomedical applications, not only due to their nontoxic character, but also because heating in dispersion at reasonable concentrations targets biologically relevant thermal ranges for magnetic hyperthermia, typically between 40 and 50°C [98, 99, 100].

Overall, Zn ferrite NPs exhibit SAR values comparable to those reported in literature [101, 102, 103, 104, 105]. It is also described that the SAR decreases with higher Zn doping than Zn_0.3_Fe_2.7_O_4_, which mirrors the behavior of saturation magnetization and fits to the herein presented doping levels, due to the structurally reduced inversion with higher nonmagnetic Zn^2+^ content [101, 106]. In general, Zn ferrite NPs display a high colloidal stability, which enables their good induction heating properties in aqueous dispersion, together with their synthetic and postsynthetic means to actively influence induction heating temperatures. Overall, while it oftentimes poses a problem to find species that display both good induction heating characteristics and colloidal stability, the herein presented NP species unite both fields. Low Zn dopings and higher SAR values could be fitting for systems that have to be heated fast without toxic Co in the system, as Zn0.3 ferrite NPs heat even better than pure Co ferrites in dispersion. High Zn dopings and hence a lower SAR could be valuable in systems relevant for magnetic hyperthermia, where critical temperature thresholds in cells must not be exceeded.

## 3 Conclusion

In conclusion, we established the scalable spray‐drying‐based synthesis route for Zn ferrite NPs (Zn_X_Fe_3−__X_O_4_) with finely tunable maximum induction heating temperatures spanning a wide thermal range, including thresholds below 100°C, in which fine‐tuning is still largely unexplored. The Zn doping level could be adjusted starting from Zn_0.3_Fe_2.7_O_4_ up to unusually high contents of Zn_0.75_Fe_2.25_O_4_ that could be confirmed by HR‐TEM and HAADF‐STEM‐EDX measurements. NPs were formed by spray‐drying the corresponding ratios of the precursor salts with TEOS to form SiO_2_‐based microparticles. Via variation of the postsynthesis annealing temperature between 1000 and 1100°C, the maximum induction heating temperatures of the resulting NPs of each composition could be precisely controlled. Increasing the annealing temperature and decreasing the Zn doping both led to higher induction heating temperatures in each case. Consequently, the induction heating performance of the NPs could be tailored between room temperature and 250°C through selection of the Zn doping and annealing conditions. A comparable trend was observed in the corresponding dispersions, where Zn_0.3_Fe_2.7_O_4_ exhibited an SAR of 369 W g^−1^, while samples with higher Zn content reached biologically relevant temperatures within less than three minutes, depending on the concentration. Notably, all aqueous dispersions displayed a high colloidal stability due to the electrostatic stabilization provided by the remaining SiO_2_ shell, leading to a beneficial combination of good dispersibility and comparably high heating rates. The reduction in maximum heating temperature with increasing Zn doping is attributed to the preferential occupation of tetrahedral sites by Zn^2+^ ions in the spinel lattice, which expands the lattice parameter and decreases both the Curie temperature and the overall magnetic moment. Conversely, higher annealing temperatures promote formation of larger NPs once the SiO_2_ matrix exceeds its glass transition temperature, resulting in higher induction heating efficiencies. Moreover, variation of the AMF for each doping level and each annealing temperature, respectively, demonstrated an additional means of fine‐tuning the heating thresholds for a given magnetic NP composition. Overall, the tunable induction heating properties of the synthesized Zn ferrite NPs stem from three different hierarchies: (i) the synthesis route (regarding the Zn doping level), (ii) the postsynthesis modification (regarding the annealing temperature), and (iii) the external AMF regulation (regarding the induction heater settings). This approach offers a versatile platform for designing magnetic NPs tailored for both high‐temperature industrial processes and low‐temperature biological applications, where the nontoxicity, abundance, and biocompatibility of Zn represent key advantages for future studies.

## 4 Experimental Section

### 4.1 Materials and Reagents

Iron(II) nitrate nonahydrate (Fe(NO_3_)_3_·9H_2_O, 96%), zinc(II) nitrate hexahydrate (Zn(NO_3_)_2_·6H_2_O, 98%), potassium hydroxide (KOH, 90%, flakes), and sodium hydroxide (NaOH, ≥ 98%, pellets) were received from Carl Roth. Iron(II) sulfate heptahydrate (FeSO_4_·7H_2_O, 99.5%) and potassium nitrate (KNO_3_, 99+%) were obtained from Acros Organics. Tetraethyl orthosilicate (SiC_8_H_20_O_4_, TEOS, 99%) was obtained from abcr. All chemicals and reagents were used without further purification. Water was deionized prior to usage. For all synthesis procedures, technical ethanol (EtOH) was used.

### 4.2 Synthesis of Zn Ferrite NPs

Ferrite NPs with different amounts of Zn doping were synthesized based on a modified spray‐drying method to obtain iron oxides introduced by Jo et al. [65]. For an initial Fe:Zn ratio of 9:1, Fe(NO_3_)_3_·9H_2_O (5.31 g, 13.14 mmol) and Zn(NO_3_)_2_·6H_2_O (0.43 g, 1.46 mmol) were dissolved in a mixture of H_2_O (21.62 g, 1.20 mol) and EtOH (27.64 g, 0.60 mol). TEOS (7.60 g, 36.48 mmol) was added dropwise to the stirred solution, inducing a color change from orange to yellow. For other Zn dopings, the molar ratio of precursor salts (Fe, Zn) to Si was kept constant at 0.4, while the Fe:Zn ratio was adjusted to 5.7:1, 4:1, and 3:1, respectively. The precursor mixture was spray‐dried in each case to yield a light brown powder (yield ∼4 g) using a Büchi Labortechnik AG spray‐dryer (B‐290 mini) connected to a dehumidifier B‐296. All spray‐drying runs were conducted with identical parameters (inlet temperature: 220°C, pump rate: ∼0.18 L h^−1^, aspirator power: 100%, resulting in a volume flow of ∼35 m^3^ h^−1^, spraying gas flow: 742 L h^−1^, outlet temperature: ∼100°C). After spray‐drying, the powders were heated at varying temperatures between 1000 and 1100°C for 4 h in a high‐temperature furnace (Nabertherm LT 5/11 with B400/B410 Controller) at a heating ramp of 300 K h^−1^, inducing a color change to reddish brown. The powder was subsequently treated with NaOH (5 m, 100 mL) at 70°C for 24 h to remove SiO_2_ residues, before the resulting dispersions were centrifuged (Hermle Z 326 K, 1 × 8000 rpm for 1 min, 2 × 12 000 rpm for 5 min) and redispersed in 30 to 40 mL of deionized water. The synthesis can be scaled up to desired yields.

### 4.3 Synthesis of FIONs

A solution of FeSO_4_·7H_2_O (10.01 g, 36.01 mmol) was prepared by dissolving it in 1620 g of deionized water and heating to reflux under a nitrogen atmosphere. Separately, KNO_3_ (25.48 g, 252.02 mmol) and KOH (6.17 g, 109.96 mmol) were dissolved in 180 g of deionized water to form the precipitating solution, which was degassed with nitrogen and maintained at 60°C. Once the FeSO_4_ solution reached reflux, the precipitating solution was added. The mixture was then refluxed under continuous nitrogen degassing for 5 h, resulting in a dark brown NP suspension. After cooling to room temperature, the NPs were collected by centrifugation (2 × 8000 rpm for 2 min, 1 × 8000 rpm for 5 min) and redispersed in 100 mL of deionized water.

### 4.4 Material Characterization

SEM characterization was conducted using a JSM‐F100 (Jeol). Samples were prepared on carbon pads (Plano) and sputtered with Pt (108SE, Cressington, 40 mA, 20 s). Morphology was examined using a secondary electron detector and an acceleration voltage of 5 kV at a working distance of 6 mm. EDX analyses were conducted with an acceleration voltage of 15 kV and a working distance of 10 mm at constant peak intensity acquisition times of 8 min.

Induction heating experiments were conducted employing the high‐frequency device Sinus 52 (Himmelwerk) with a two‐wind coil (Cu) at a frequency of 1419 kHz and AC magnetic fields with a maximum amplitude of 500 Oe for varying durations (60 to 900 s). The magnetic field amplitudes were simulated in collaboration with the Himmelwerk company. The temperature at the surface of magnetic samples was measured with a CTlaser LTF pyrometer (Optris). For the induction heating of dried agglomerated NPs, 30 mg of the sample were transferred to a 75 µL crucible (Al_2_O_3_) that was placed in the center of the coil. For the induction heating of NP dispersions, 3 mL of dispersions with varying concentrations were transferred to an open vial that was also placed in the center of the coil. The magnetic field amplitude was adjusted manually to different values (50 to 500 Oe) depending on the experiment.

Magnetization curves of magnetic NP and SP samples were obtained using a superconducting quantum interference device (SQUID) MPMS3 (Quantum Design Inc.) with which the applied magnetic field was cycled from 30 to 30 kOe employing a step rate of 50 Oe s^−1^. Detailed analyses were carried out with the same device by cycling the applied field from 500 to 500 Oe with a step rate of 2 Oe s^−1^. The temperature for all measurements was set to 300 K. All samples were measured in pressed form in a cylindrical capsule introduced to a brass sample holder. The sample holder contribution is automatically accounted for in the software, and the cylindrical geometry of the pressed samples ensures that demagnetization and geometric effects are negligible. Consistent sample preparation and measurement orientation further minimize any geometry‐related artifacts. ZFC curves were measured by cooling the sample from room temperature to 30 K without any magnetic field present, before applying a field of 100 Oe and measuring while warming up to 400 K. FC curves were measured by heating the sample to 400 K, then applying a field of 100 Oe and cooling down to 30 K, before measuring while warming up to 400 K. For both procedures, the sweep rate was 3 K min^−1^, the peak amplitude 3 mm, and the averaging time 3 s. The *T*_B_ was estimated from the maximum of the ZFC curve in each case.

TEM images were recorded with an LEO EM 900 microscope (Carl Zeiss AG, Oberkochen, Germany) using an acceleration voltage of 80 kV. For sample preparation, diluted aqueous NP dispersions were casted on a carbon‐coated copper TEM grid. NP size distributions were averaged over 100 NPs from TEM images by manual evaluation using ImageJ.

TGA measurements for Curie temperature determination were conducted on 5 to 10 mg of dried NP weight with an attached static high‐energy magnet (Netzsch, TGA209F1E93.100‐00) on a TG 209 F1 Libra (Netzsch) by heating the samples in a nitrogen atmosphere (N_2_: 50 mL min^−1^) with a heating rate of 30 K min^−1^ in the temperature range from 30 to 1000°C. For a more reliable measurement, a nonmagnetic counterweight in the form of SiO_2_ (sand, Carl Roth) was utilized to refrain from mass falsifications induced by lifting of the crucible lid, which is otherwise caused by the experimental setup.

XRD was recorded using a D8 Advance diffractometer (Bruker) equipped with a Lynxeye XE‐T detector employing Cu Kα radiation (*λ* = 0.154056 nm). Measurements were conducted in the 2θ range from 20° to 80° at 0.02° increments. Indication of reflections was performed based on the International Centre for Diffraction Data PDF‐4.

For XRD measurements with Rietveld refinement, the powders were prepared via front loading method into a silicon single crystal cavity sample holder. Three independent preparations of each sample were measured. All XRD measurements were performed at a D8 diffractometer (Bruker AXS, Karlsruhe, Germany) equipped with a LYNXEYE detector. The following measurement parameters were applied: Range 10°–85° 2θ; step size 0.0236° 2θ, integration time 0.8 s; radiation: Cu K_α_; generator settings: 40 mA, 40 kV; divergence slit: 0.3°. Quantitative evaluation of the diffraction patterns was performed with software TOPAS V7 (Bruker AXS, Karlsruhe, Germany). The structure models used for the refinement were obtained from the Inorganic Crystal Structure Database (ICSD; FIZ Karlsruhe, Germany) (Table S11). Refined parameters were scale factors, lattice parameters, and crystallize size (Lorentz contribution, Cry size L and Gauss contribution, Cry Size G where appropriate). The background was modelled with a Chebychev polynomial of fourth order.

DLS measurements were conducted on a Zetasizer Nano (Malvern Panalytical) at 25°C monitoring the scattering signal in a backscattering angle (173°). The reported data represent a mean value of 3 individual measurements, each containing 10 individual runs.

For HR‐TEM, HAADF‐STEM, STEM‐EDX, and SAED measurements, the corresponding NPs were drop‐cast onto an ultrathin carbon on Lacey carbon copper TEM grid and naturally dried in ambient conditions. Images were taken with a FEI Titan Themis 300 transmission electron microscope operated at 300 kV in TEM mode and STEM mode (probe current: 150 pA). STEM‐EDX was performed using a Super‐X detector. To reduce contamination, samples were plasma‐cleaned for 5 s (Fischione Model 1020) and beam showered for 15 min.

The Zn and Fe contents in respective Zn ferrites were determined by ICP‐OES using a Ciros CCD (Spectro Analytical Instruments GmbH) and an Optima 8300 (PerkinElmer). The solid samples were dissolved in concentrated HCl:HNO_3_:HF in a volumetric ratio of 3:1:1 in 100 mL, employing microwave heating to 220°C for 40 min.

Photographs were taken with a digital camera (Fujifilm X‐T30 II).

## Author Contributions

L. Luthardt (Conceptualization: Lead; Investigation: Lead; Methodology: Lead; Data curation: Lead; Funding acquisition: Equal; Supervision: Lead; Validation: Lead; Visualization: Lead; Writing – original draft: Lead; Writing – review and editing: Lead), S. Müssig (Conceptualization: Equal; Methodology: Equal; Supervision: Equal, Writing – review and editing: Equal; Visualization: Equal), K. Hurle (Investigation: Equal, Data curation: Equal; Writing – review and editing: Equal), A. Zink (Investigation: Equal, Data curation: Equal), X. Zhou (Investigation: Equal, Data curation: Equal, Writing – review and editing: Equal), B. Apeleo Zubiri (Investigation: Equal, Data curation: Equal, Writing – review and editing: Equal), E. Spiecker (Investigation: Equal, Writing – review and editing: Equal), K. Mandel (Conceptualization: Lead; Funding acquisition: Lead; Project administration: Lead; Resources: Lead; Supervision: Equal, Writing – review and editing: Equal).

## Funding

Bundesministerium für Forschung, Technologie und Raumfahrt (BMFTR, AdRecBat No. 03XP0518C), Deutsche Forschungsgemeinschaft (DFG, German Research Foundation, Project‐ID – 416229255, CRC1411), European Union (ERC, SmartRust, 101123921), and German Federal Environmental Foundation (DBU).

## Conflicts of Interest

The authors declare no conflicts of interest.

## Supporting information




**Supporting File**: smll72808‐sup‐0001‐SuppMat.docx.

## Data Availability

The data that support the findings of this study are openly available at Zenodo at DOI https://doi.org/10.5281/zenodo.18670440.
