# Supplementary material for: Tailorable Curie Temperature in Zinc Ferrite Nanoparticles With Finely Tunable Induction Heating Profiles Between Room Temperature and 250°C
Source: Small. 2026 Feb 23;22(21):e13314. doi: 10.1002/smll.202513314 (PMC13081108; doi:10.1002/smll.202513314)
Supplement: Supplementary file 1 — Supporting File: smll72808‐sup‐0001‐SuppMat.docx. [file SMLL-22-e13314-s001.docx]

# Supporting Information

**Tailorable Curie Temperature in Zinc Ferrite Nanoparticles with Finely Tunable Induction Heating Profiles Between Room Temperature and 250 °**C

Leoni Luthardt, Stephan Müssig, Katrin Hurle, Andreas Zink, Xin Zhou, Benjamin Apeleo Zubiri, Erdmann Spiecker, and Karl Mandel^*^


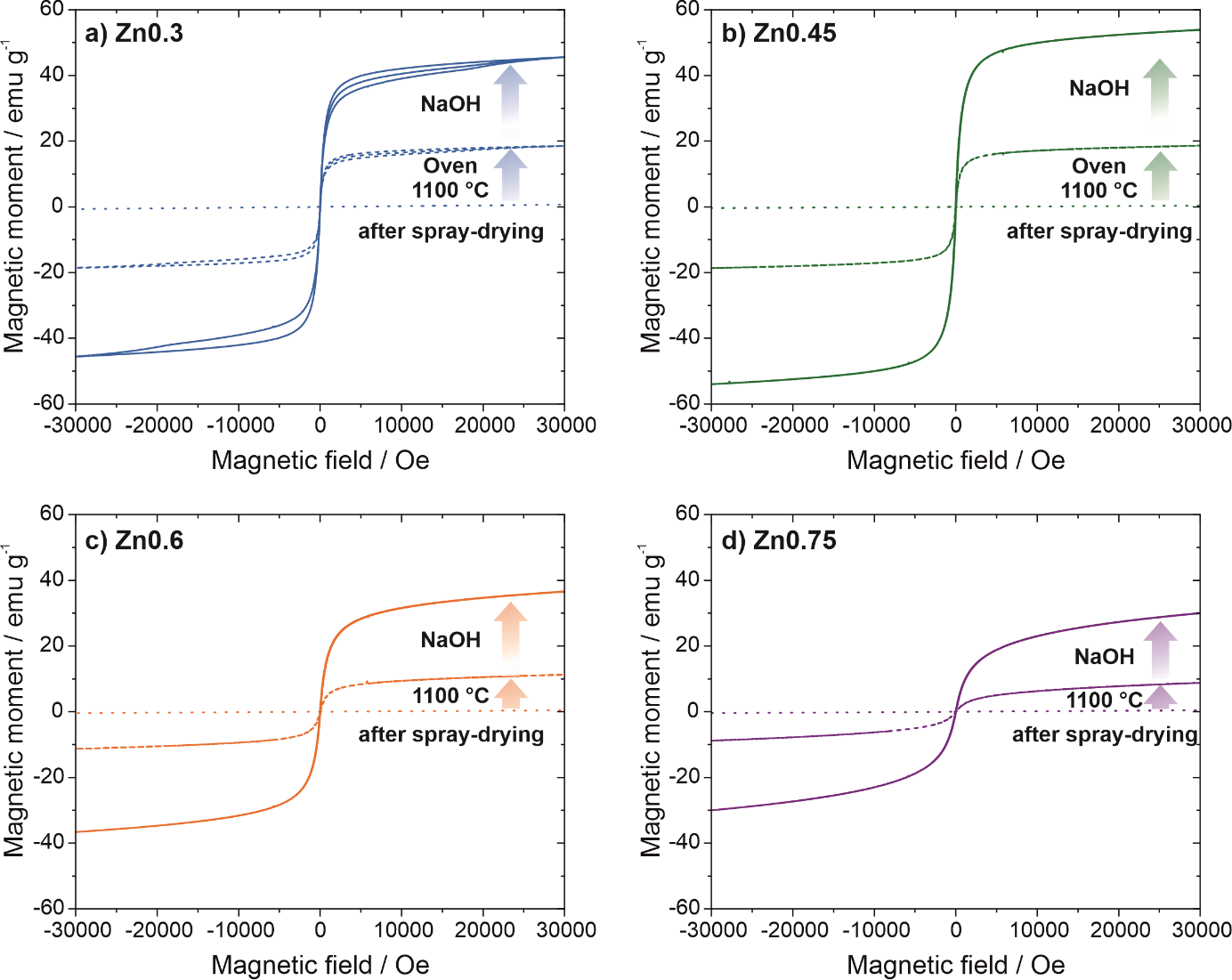


**Figure S1.** Magnetization curves of varying Zn dopings in Zn ferrite NPs at different stages of the synthesis process, shown for molar Fe to Zn ratios of 9:1 (a), 5.7:1 (b), 4:1 (c), and 3:1 (d). Dotted lines symbolize the magnetic state directly after spray-drying, dashed lines that after thermal annealing at high temperatures, and solid lines that after caustic treatment. In each case, no magnetic moment was measurable after spray-drying, whereas a magnetic moment evolved after thermal treatment at 1100 °C, indicating the formation of magnetic NPs. The magnetization per mass was increased in each case after treatment with NaOH, as the non-magnetic SiO_2_ matrix is dissolved. Magnetization values are given in emu per gram of the measured powder sample mass.


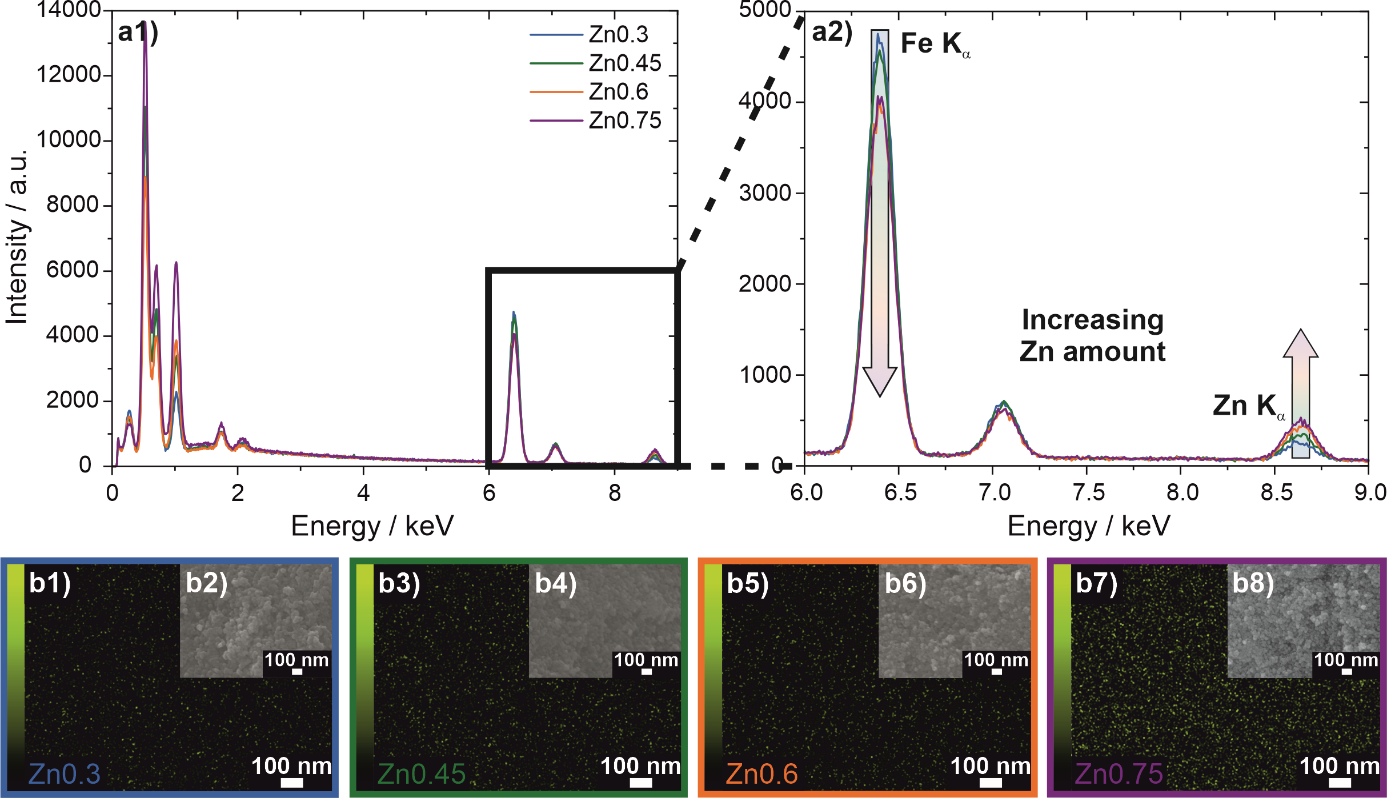
**Figure S2.** EDX analysis of differently doped Zn ferrite NPs. a) EDX spectrum reveals an increasing Zn K_α_ and a decreasing Fe K_α_ peak for an increasing Zn share, indicating that the higher Zn amount is incorporated into the ferrite structure. b) Qualitative count maps reveal Zn (indicated in green) present in every NP sample independent of doping, for Zn0.3 (1,2), Zn0.45 (3,4), Zn0.6 (5,6), and Zn0.75 (7,8) NPs.

**Table S1.** Intensities of Fe K_α_ (6.39 keV) and Zn K_α_ (8.64 keV) peaks and the corresponding ratios for different Zn dopings in Zn ferrite NPs, taken from EDX spectra.

|  | **Intensity of Fe K_α_ (6.39 keV) / a.u.** | **Intensity of Zn K_α_ (8.64 keV) / a.u.** | **Ratio Fe K_α_/Zn K_α_ / a.u.** |
| --- | --- | --- | --- |
| **Zn0.3** | 4754 | 239 | 0.05 |
| **Zn0.45** | 4482 | 323 | 0.07 |
| **Zn0.6** | 3899 | 426 | 0.11 |
| **Zn0.75** | 4069 | 531 | 0.13 |

**Table S2.** ICP-OES data obtained for NPs of each molar Fe/Zn ratio employed during the spray-drying synthesis, each annealed at 1100 °C. With decreasing initial Fe/Zn molar ratio, the Zn share of the final NPs is increasing and the Fe share is decreasing. The final magnetite sum formulas for each doping closely correspond to the calculated theoretical values. Data is obtained from three individual measurements of the same sample.

| **Molar ratio Fe/Zn** | **9:1** | **5.7:1** | **4:1** | **3:1** |
| --- | --- | --- | --- | --- |
| **Zn content / wt%** | **9.18±0.60** | **12.01±0.88** | **14.85±0.46** | **19.12±0.42** |
| Theoretical Zn content / wt% | 8.37 | 12.48 | 16.53 | 20.54 |
| **Fe content / wt%** | **54.61±1.53** | **55.46±3.27** | **48.98±1.32** | **47.16±0.75** |
| Theoretical Fe content / wt% | 64.33 | 60.37 | 56.49 | 52.64 |
| Ratio Fe/Zn | 5.95:1 | 4.62:1 | 3.30:1 | 2.47:1 |
| **Magnetite sum formula** | **Zn_0.38_Fe_2.62_O_4_** | **Zn_0.47_Fe_2.53_O_4_** | **Zn_0.62_Fe_2.38_O_4_** | **Zn_0.77_Fe_2.23_O_4_** |
| **Theoretical magnetite sum formula** | **Zn_0.3_Fe_2.7_O_4_** | **Zn_0.45_Fe_2.55_O_4_** | **Zn_0.6_Fe_2.4_O_4_** | **Zn_0.75_Fe_2.25_O_4_** |

**Table S3.** Crystallite sizes of individual Zn ferrite NP species as obtained from Rietveld refinement of three independently prepared XRD samples of each doping, annealed at 1100 °C, given in nm (mean±SD). With higher Zn share, the crystallite size increases.

|  | **Crystallite size of magnetite / nm** |
| --- | --- |
| **Zn0.3** | 7.20±0.04 |
| **Zn0.45** | 6.89±0.03 |
| **Zn0.6** | 7.26±0.10 |
| **Zn0.75** | 8.52±0.02 |

**Table S4.** Shares of different iron oxide phases as obtained from Rietveld refinement of three independently prepared XRD samples of each different Zn doping, annealed at 1100 °C, given in wt% (mean±SD). With higher Zn share, a pure magnetite structure is obtained, whereas in lower Zn-doped samples, ε-Fe_2_O_3_ or hematite fractions remain.

|  | **Magnetite share / wt%** | **Hematite share / wt%** | **ε-Fe_2_O_3_ share / wt%** |
| --- | --- | --- | --- |
| **Zn0.3** | 67.0±0.4 | 0 | 33.0±0.4 |
| **Zn0.45** | 96.3±0.4 | 3.8±0.4 | 0 |
| **Zn0.6** | 100 | 0 | 0 |
| **Zn0.75** | 100 | 0 | 0 |


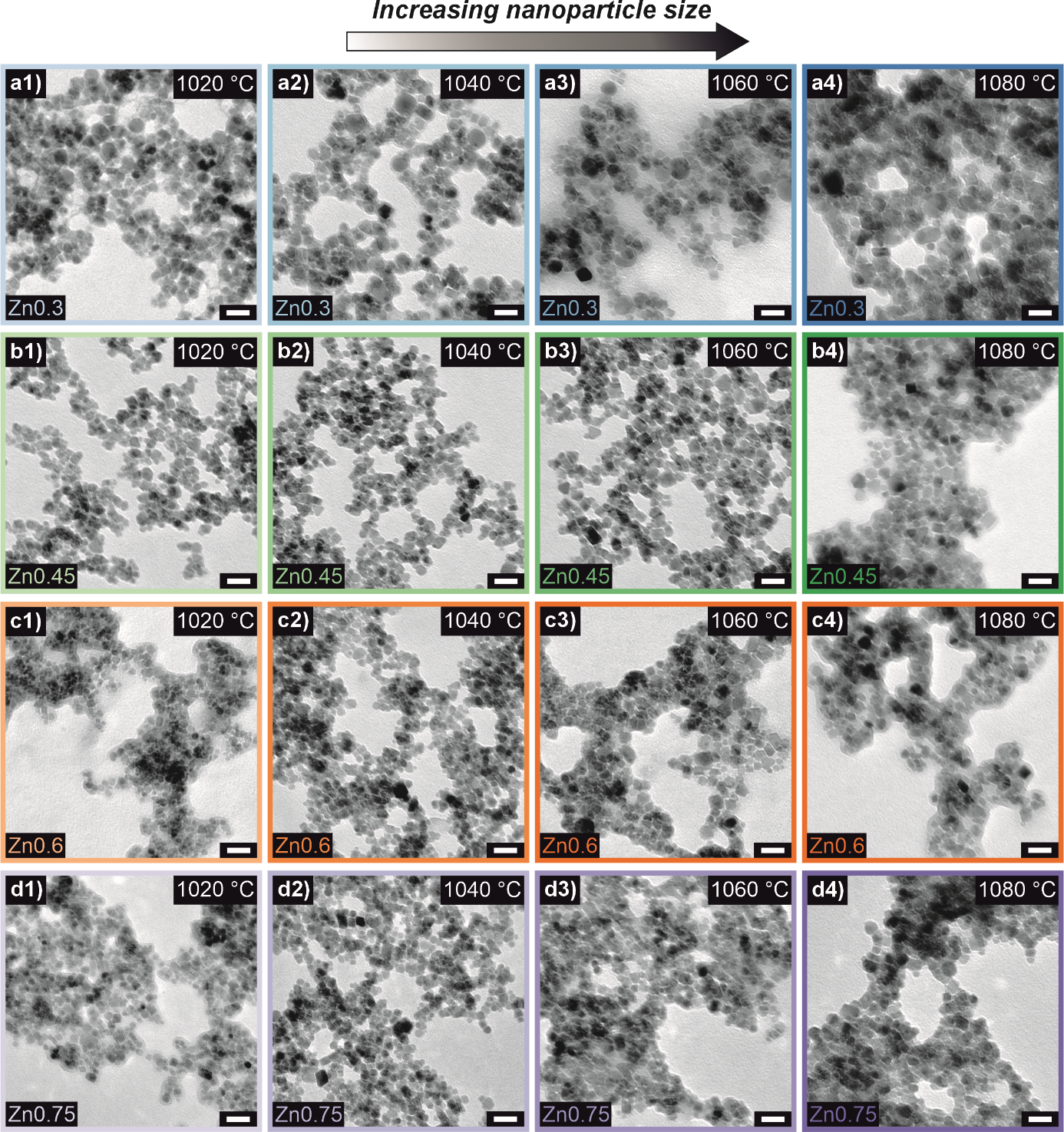


**Figure S3.** TEM images for Zn0.3 (a), Zn0.45 (b), Zn0.6 (c), and Zn0.75 (d) NPs, annealed at 1020 (1), 1040 (2), 1060 (3), and 1080 °C (4), respectively. All images reveal an increasing NP size with increasing annealing temperature post-synthesis. Scale bars correspond to 25 nm.


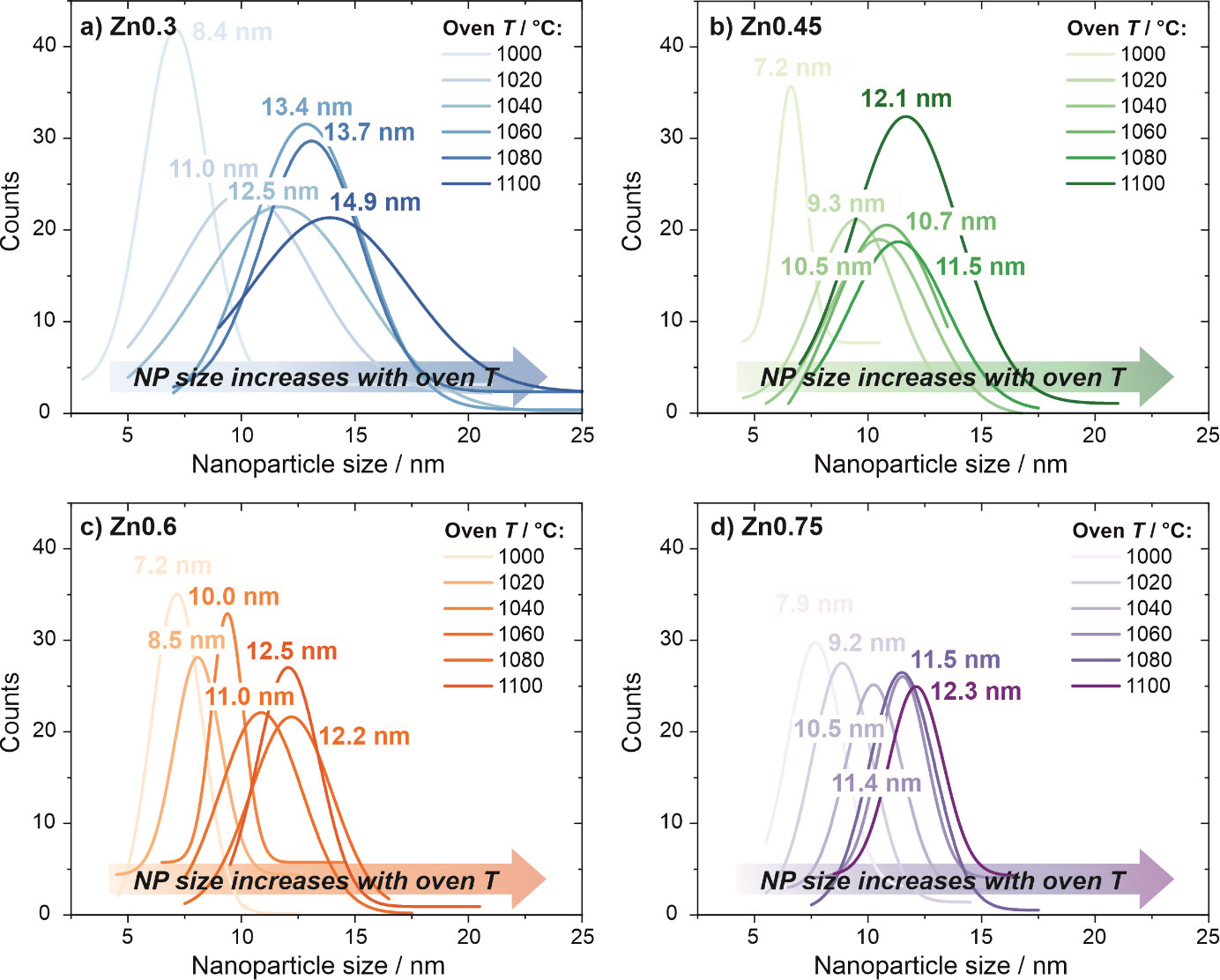


**Figure S4.** NP size distributions for Zn0.3 (a), Zn0.45 (b), Zn0.6 (c), and Zn0.75 (d) NPs, annealed at different temperatures between 1000 and 1100 °C. In each case, the NP size increases with increasing oven temperature post-spray-drying. NP sizes were determined over 100 individual NPs for each doping and each temperature treatment as read out from TEM images.


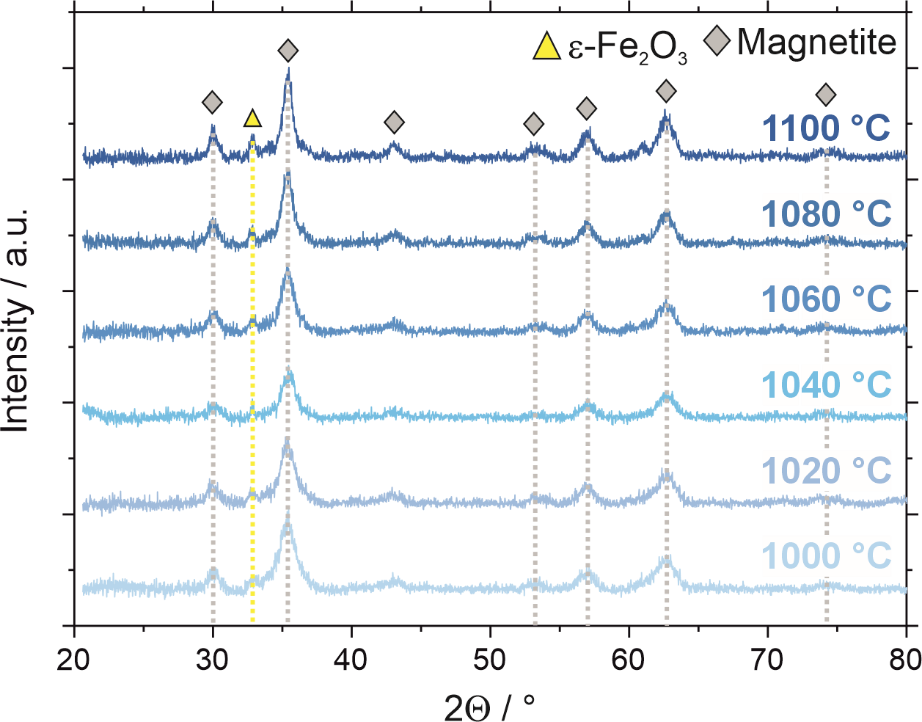


**Figure S5.** XRD of Zn0.3 NPs annealed at different temperatures between 1000 and 1100 °C. A magnetite/maghemite crystal structure is maintained independent of oven temperature, even though reflex intensity and therefore crystallinity are increasing with increasing temperature. An ε-Fe_2_O_3_ fraction is apparent for all thermal treatments.

**Table S5.** Lattice parameter a of magnetite in different Zn-doped samples, each annealed at 1100 °C. The data were obtained by Rietveld refinement and are presented as mean ± SD (n = 3). With higher Zn share, the a parameter of magnetite is increased.

|  | ***a* parameter of magnetite / Å** |
| --- | --- |
| **Zn0.3** | 8.3974±0.0055 |
| **Zn0.45** | 8.4062±0.0001 |
| **Zn0.6** | 8.4204±0.0026 |
| **Zn0.75** | 8.4298±0.0010 |

*
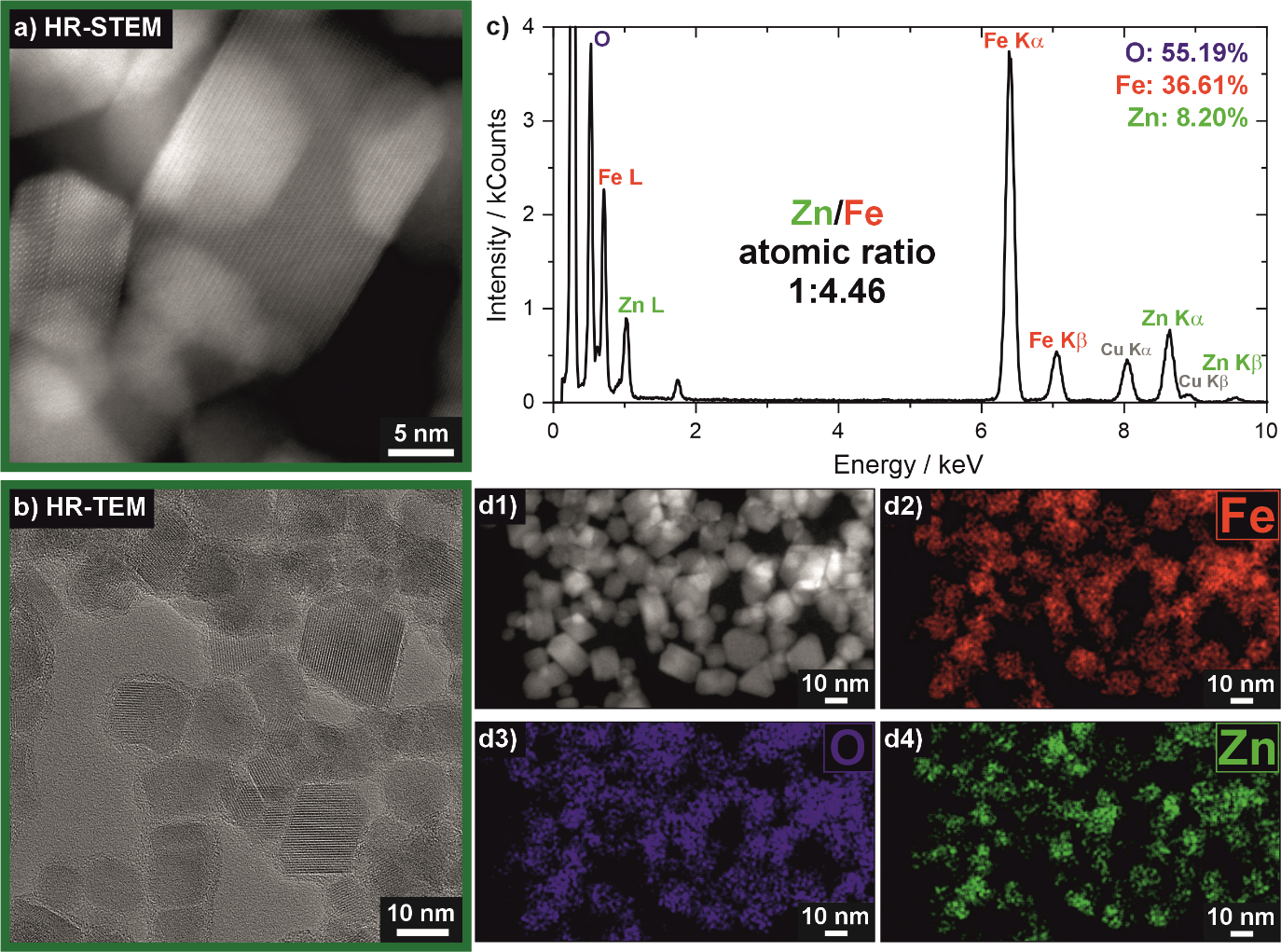
*

**Figure S6.** Structural investigation of Zn_0.45_Fe_2.55_O_4_ NPs by HR-STEM (a) and HR-TEM (b), displaying a spinel lattice without secondary or amorphous phases. STEM-EDX (c, d) showcases an even spatial distribution of Zn throughout the NPs, with a calculated Zn:Fe atomic ratio of 1:4.46, which is less than the nominal stoichiometry (1:5.67) to a minor extent, but still matches the trend well. The X-ray peaks at ~8 keV, ~8.9 keV are related to Cu-Kα, and Cu-Kβ, mainly contributed by the Cu TEM grid. The X-ray peak at ~1.7 keV is related to Si, mainly contributed from the detector. The huge peak at ~0.3 keV is attributed to C.

**Table S6.** Lattice parameter a of magnetite in different Zn-doped samples, each annealed at 1100 °C, determined from SAED. The lattice constant increased with Zn doping, in this case by 0.9% between Zn0.45 and Zn0.75.

|  | ***a* parameter / Å** |
| --- | --- |
| **Zn0.45** | 8.3496±0.0159 |
| **Zn0.75** | 8.4252±0.0108 |

*
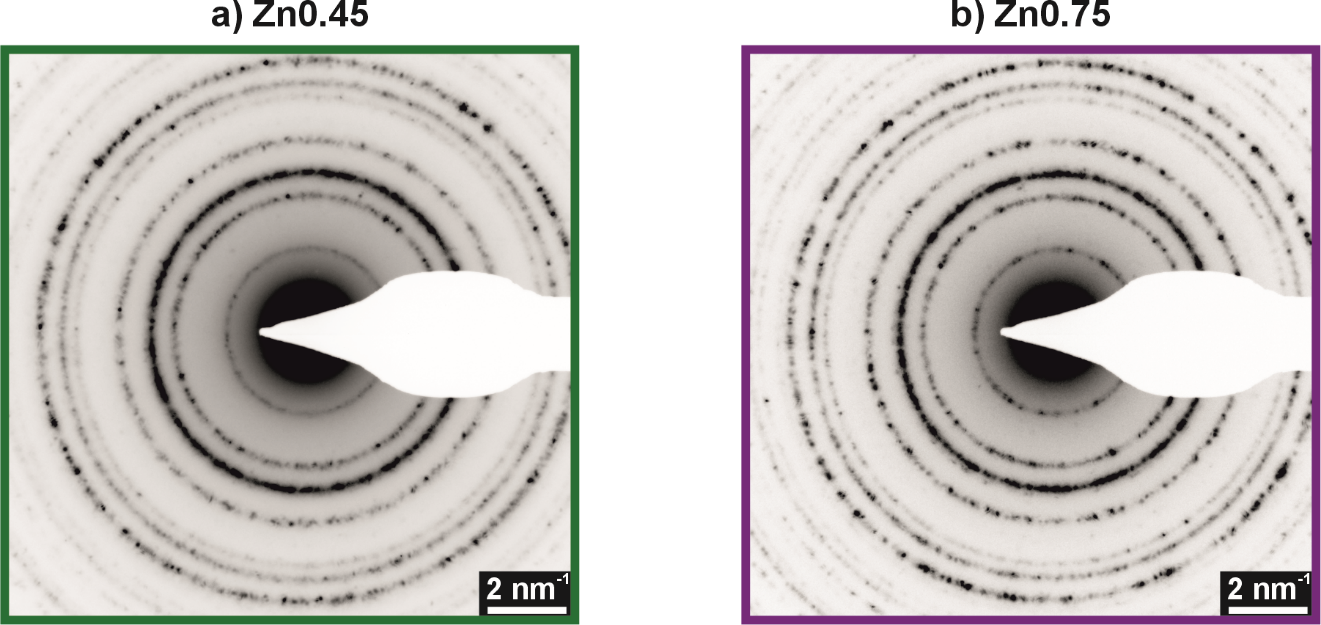
*

**Figure S7.** Raw SAED pattern for Zn0.45 (a) and Zn0.75 (b) NPs, from which the a parameter was obtained.


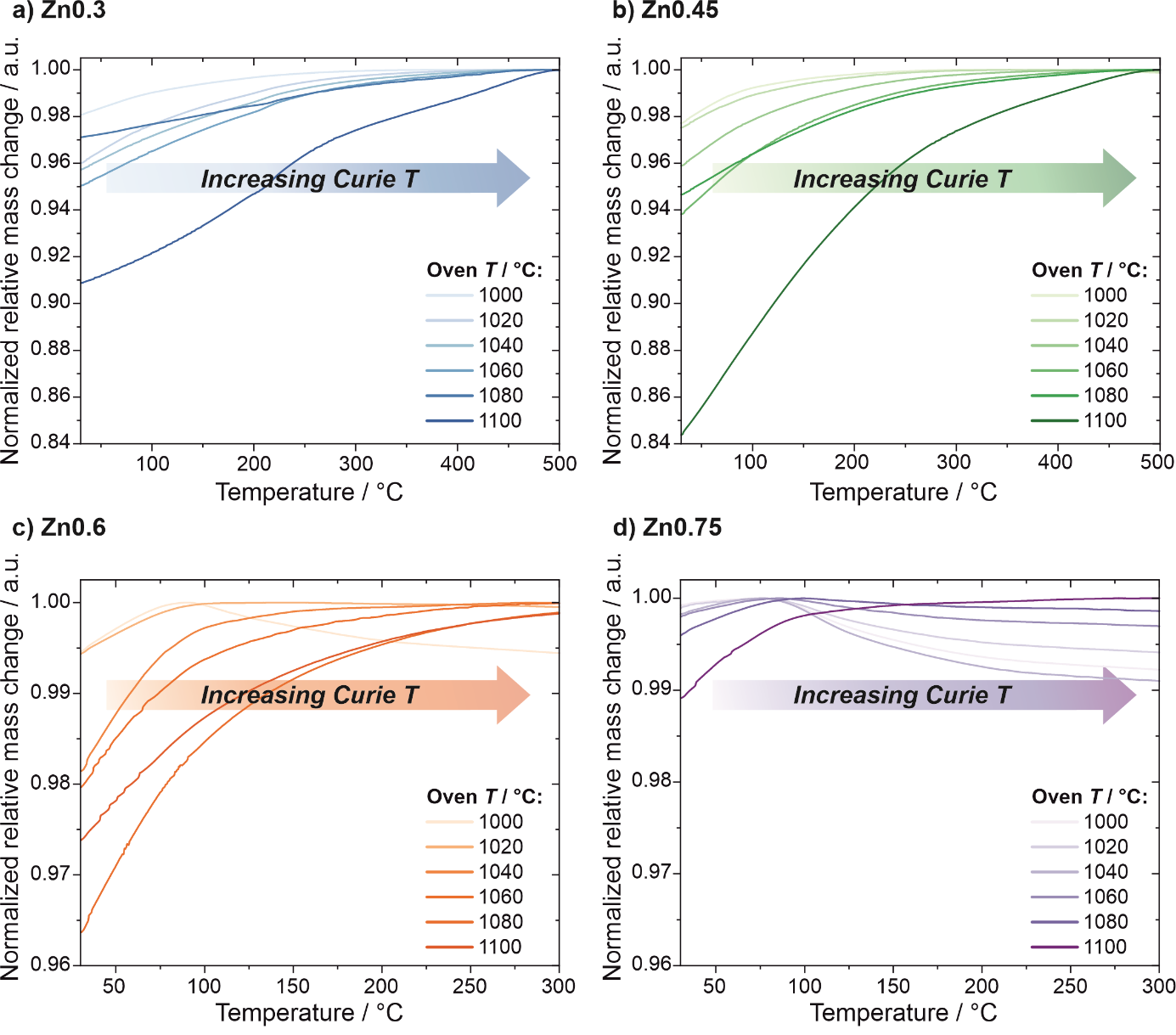


**Figure S8.** Curie temperatures as determined by static magnet-supported TGA measurements for Zn0.3 (a), Zn0.45 (b), Zn0.6 (c), and Zn0.75 (d) NPs at all tested oven annealing temperatures. With increasing Zn doping, the Curie temperature is reduced, whereas for differently annealed samples of the same doping, the Curie temperature increases with increasing oven temperature.

**Table S7.** Induction heating rates given in K s^-1^ for every Zn doping at every respective annealing temperature, as determined with a linear fit over the first 5 s of the respective induction heating curve recorded at an AMF amplitude of 500 Oe and a frequency of 1.4 MHz.

|  | **1000 °C** | **1020 °C** | **1040 °C** | **1060 °C** | **1080 °C** | **1100 °C** |
| --- | --- | --- | --- | --- | --- | --- |
| **Zn0.3** | 9.0 | 9.9 | 10.0 | 15.2 | 22.3 | 40.2 |
| **Zn0.45** | 2.6 | 4.9 | 7.1 | 11.0 | 18.6 | 23.2 |
| **Zn0.6** | 0.7 | 1.3 | 2.4 | 2.9 | 4.5 | 8.4 |
| **Zn0.75** | 0.5 | 0.7 | 0.7 | 1.0 | 1.5 | 3.0 |


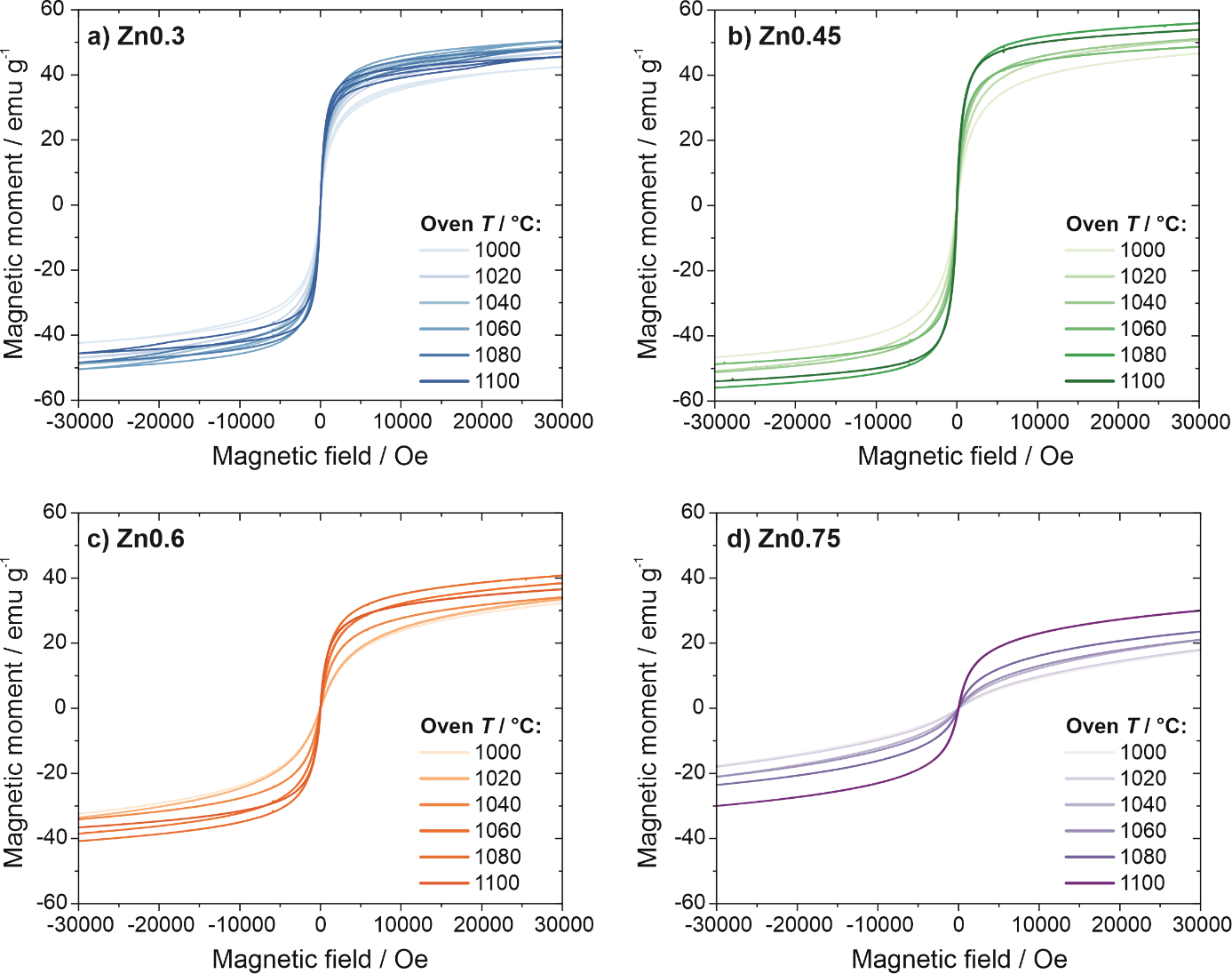


**Figure S9.** VSM measurements of Zn0.3 (a), Zn0.45 (b), Zn0.6 (c), and Zn0.75 (d) NPs recorded between -30 and 30 kOe at different oven annealing temperatures, respectively. Magnetization values are given in emu per gram of the measured powder sample mass.

**Table S8.** Mean T_B_ and estimated upper T_B_ limit for all Zn dopings, each annealed at 1000 and 1100 °C. The mean T_B_ was determined from the maximum of the ZFC, while the upper T_B_ limit was estimated from the temperature at which FC and ZFC curves converge. Due to the polydispersity of all samples, T_B_ distribution is rather large. At lower dopings, the upper limit is above 400 K and therefore outside of the measurement range, hence, the upper limit was estimated in certain range.

| **Sample** | **Annealing *T* / °C** | **Mean *T_B_* / K** | **Upper *T_B_* limit / K** |
| --- | --- | --- | --- |
| **Zn0.3** | 1000 | 55 | ~ 400 |
|  | 1100 | 201 | ~ 450 – 500 |
| **Zn0.45** | 1000 | 84 | ~ 400 |
|  | 1100 | 133 | ~ 410 – 450 |
| **Zn0.6** | 1000 | 56 | ~ 290 |
|  | 1100 | 89 | ~ 400 |
| **Zn0.75** | 1000 | 38 | ~ 180 |
|  | 1100 | 65 | ~ 295 |

*
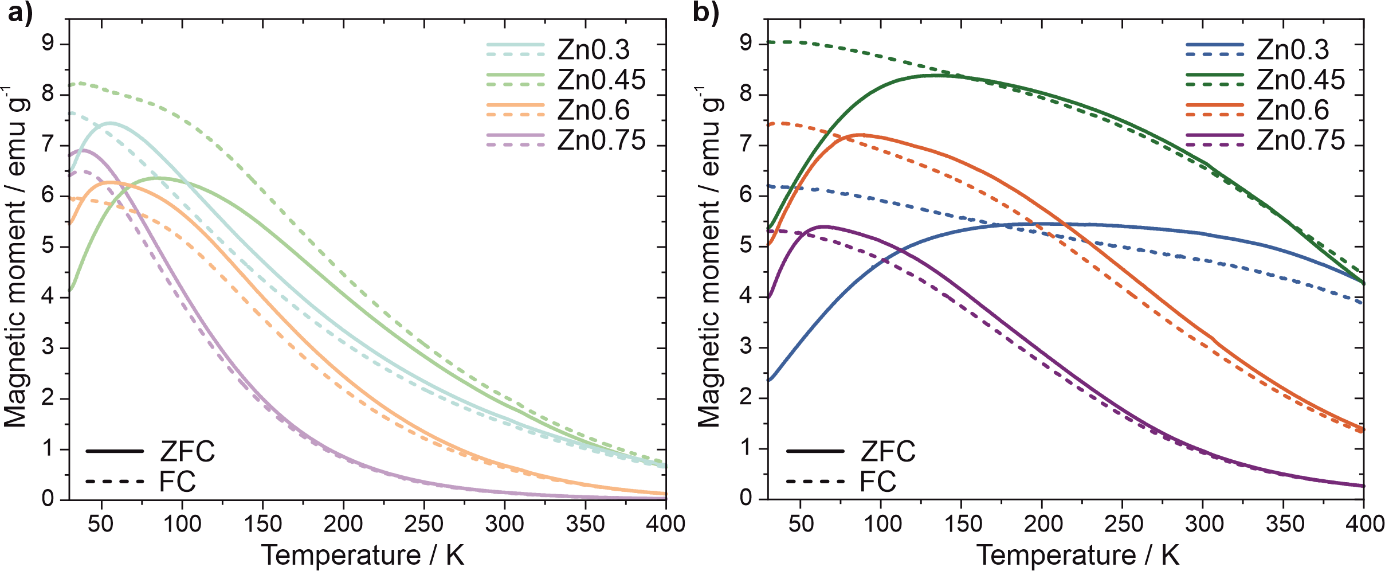
*

**Figure S10.** ZFC/FC curves of differently doped Zn ferrite NPs, annealed at 1000 (a) and 1100 °C (b), respectively. ZFC curves were recorded by zero field cooling to 30 K, then applying a field of 100 Oe and measuring while warming up to 400 K. FC curves were corded by field cooling with 100 Oe to 30 K, then measuring while warming up to 400 K. The mean T_B_ was determined from the maximum of the ZFC curve, while the convergence of ZFC and FC curves in each case present an upper limit for the T_B_ distribution in polydisperse samples.

For all samples, the maximum of the ZFC curve is taken as the mean *T_B_* corresponding to the temperature at which the largest fraction of particles becomes unblocked and can align with the applied magnetic field. Owing to the polydisperse nature of the NP ensembles, NPs with smaller volumes unblock at lower temperatures, while larger NPs remain blocked up to higher temperatures. The temperature at which the ZFC and FC curves converge therefore defines an upper limit of the *T_B_* distribution, indicating that all NPs, including the largest ones, have become superparamagnetic.^[1]^

However, for some samples, irregularities are observed in the FC curves. In particular, samples annealed at 1000 °C exhibit a FC magnetization that is lower than the corresponding ZFC magnetization over a certain temperature range, with the two curves intersecting at lower temperatures. This behavior, not unknown in literature,^[2]^ can be attributed to the broad size distribution of these samples. During field cooling, larger particles with higher blocking temperatures become blocked at relatively high temperatures, freezing their magnetic moments along random, non-optimal easy-axis orientations. As a result, these particles cannot fully align with the applied field at lower temperatures, leading to a reduced contribution to the FC magnetization and causing the FC curve to lie below the ZFC curve in this temperature range. In contrast, during the ZFC measurement, these larger particles unblock upon heating and are able to align efficiently with the applied field once they become thermally mobile, resulting in a higher ZFC magnetization. At low temperatures, smaller particles with lower blocking temperatures align efficiently during field cooling and remain aligned as the temperature is decreased, which leads to the FC magnetization exceeding the ZFC magnetization in this regime. At intermediate temperatures, the ZFC and FC curves intersect as the relative contributions of these particle populations shift, with larger particles remaining blocked while smaller particles begin to unblock. This crossing behavior therefore reflects the polydisperse nature of the sample and the coexistence of particle populations with different blocking temperatures. Dipolar interactions and size-dependent surface spin disorder are expected to further enhance these effects.^[3]^


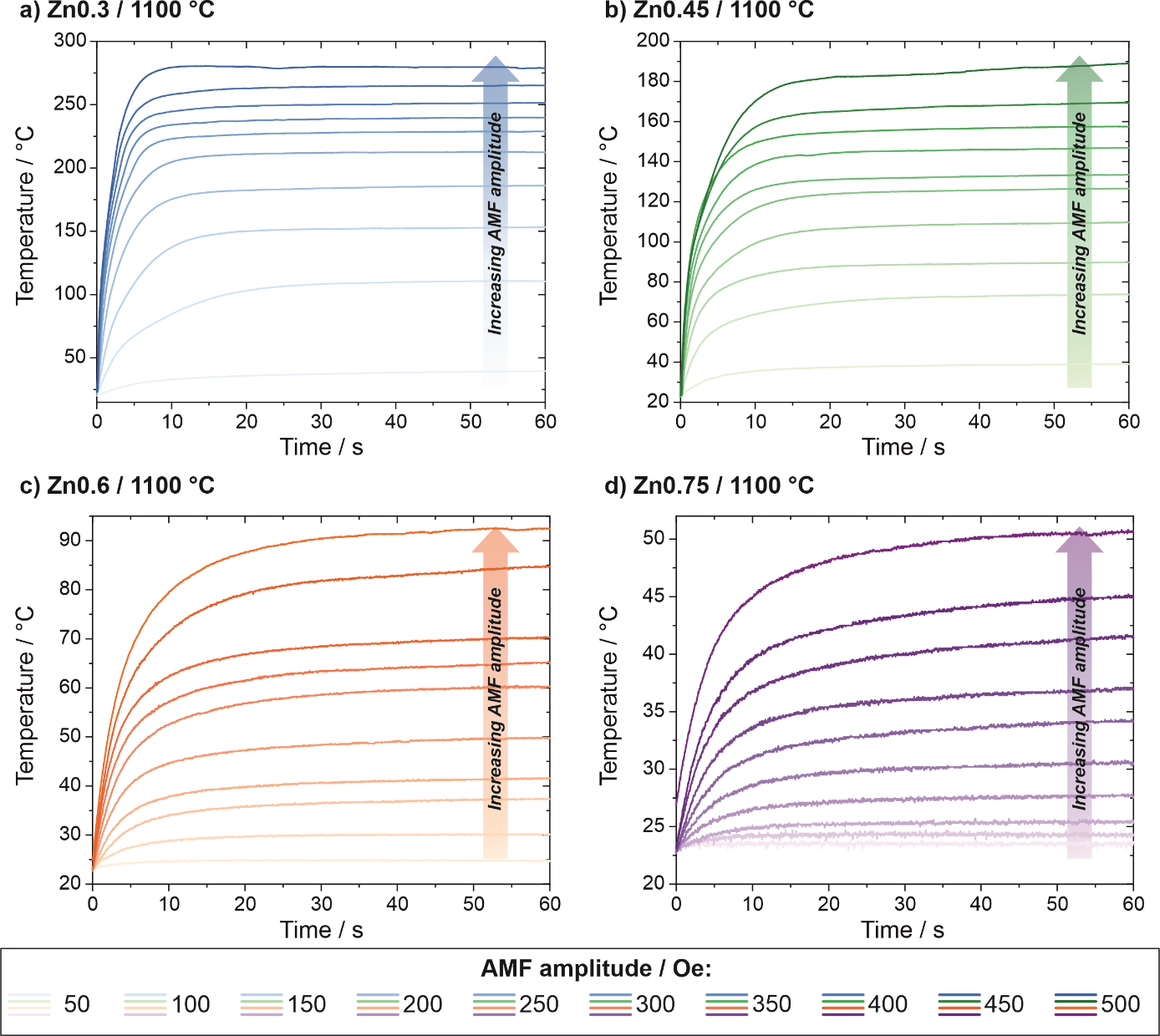


**Figure S11.** Induction heating curves of Zn0.3 (a), Zn0.45 (b), Zn0.6 (c), and Zn0.75 (d) NPs, annealed at 1100 °C each, recorded at different AMF amplitudes between 50 and 500 Oe, respectively, over a time scale of 60 s. For each doping, the temperature threshold during induction heating can be fine-tuned by actively influencing the AMF amplitude. All induction heating curves were recorded at a frequency of 1.4 MHz.


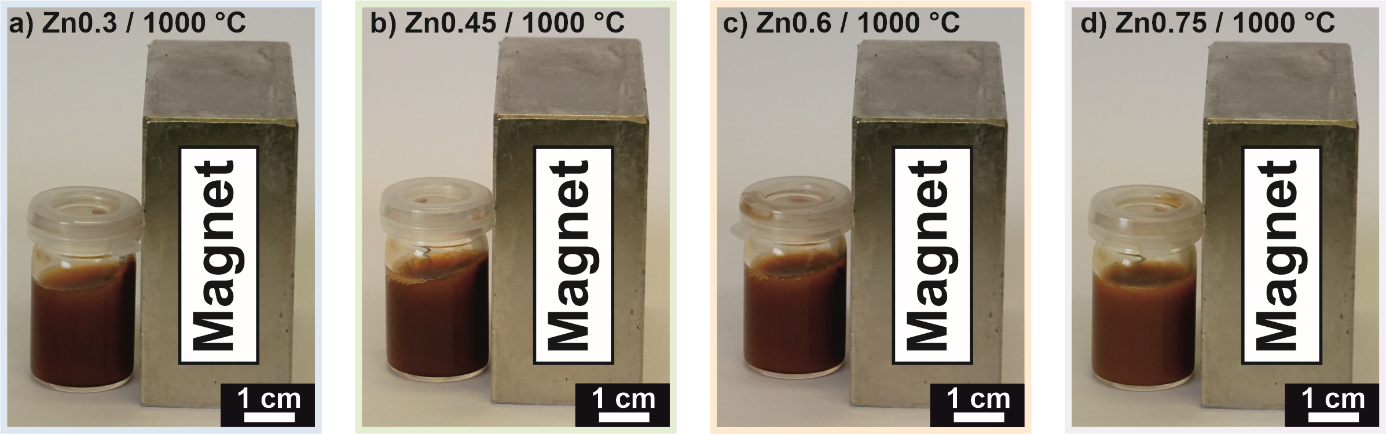


**Figure S12.** Colloidal stability of aqueous Zn ferrite dispersions of Zn0.3 (a), Zn0.45 (b), Zn0.6 (c), and Zn0.75 (d) NPs, each annealed at 1000 °C, when placed next to a static magnet with a field of approximately 4 kOe.


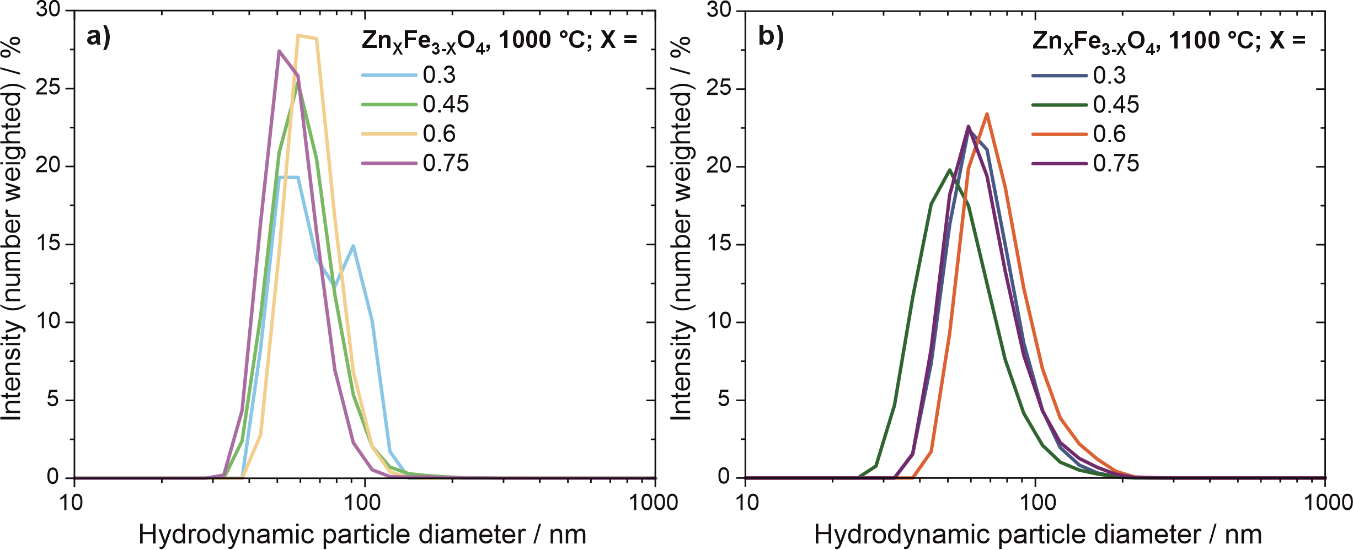


**Figure S13.** Hydrodynamic particle diameters of different Zn ferrite NPs annealed at 1000 (a) and 1100 °C (b), respectively, as measured by DLS. Data shows comparable hydrodynamic sizes of the NP types below 100 nm, in accordance with their colloidal stability and the remaining SiO_2_ shell.

**Table S9.** Mean hydrodynamic nanoparticle diameters of Zn ferrite NPs annealed at 1000 and 1100 °C, respectively, with values given in nm obtained from corresponding DLS measurements.

|  | **Mean hydrodynamic nanoparticle diameter / nm** | |
| --- | --- | --- |
|  | **Annealed at 1000 °C** | **Annealed at 1100 °C** |
| **Zn0.3** | 70 | 68 |
| **Zn0.45** | 63 | 57 |
| **Zn0.6** | 67 | 77 |
| **Zn0.75** | 57 | 68 |


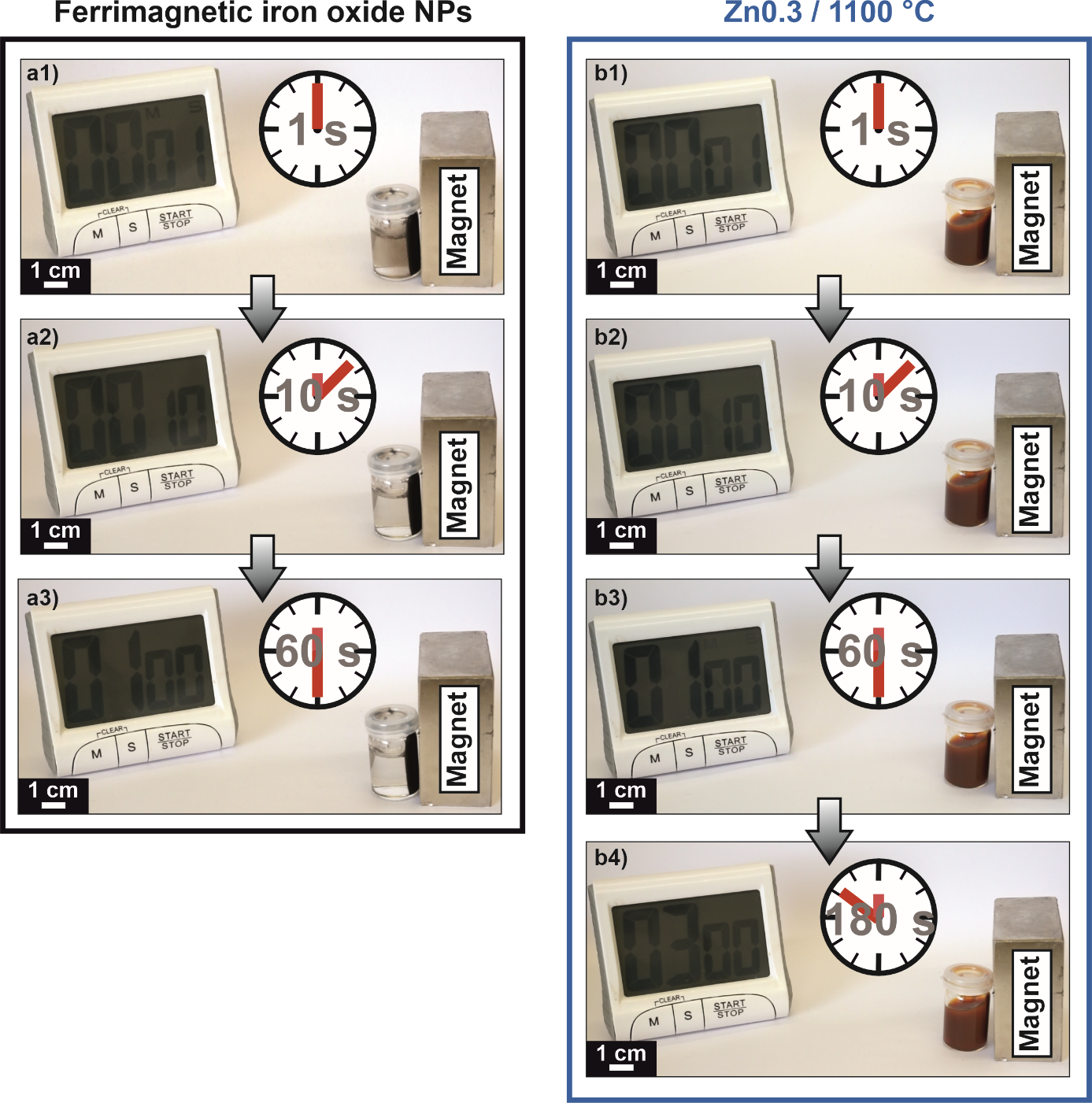


**Figure S14.** Colloidal stability over time when comparing ferrimagnetic iron oxide NPs (FIONs) (a) and Zn0.3 ferrite NPs annealed at 1100 °C (b). While FIONs are already separated after 1 s (a1), Zn ferrites stay stable in aqueous dispersion even after 3 min (b4).


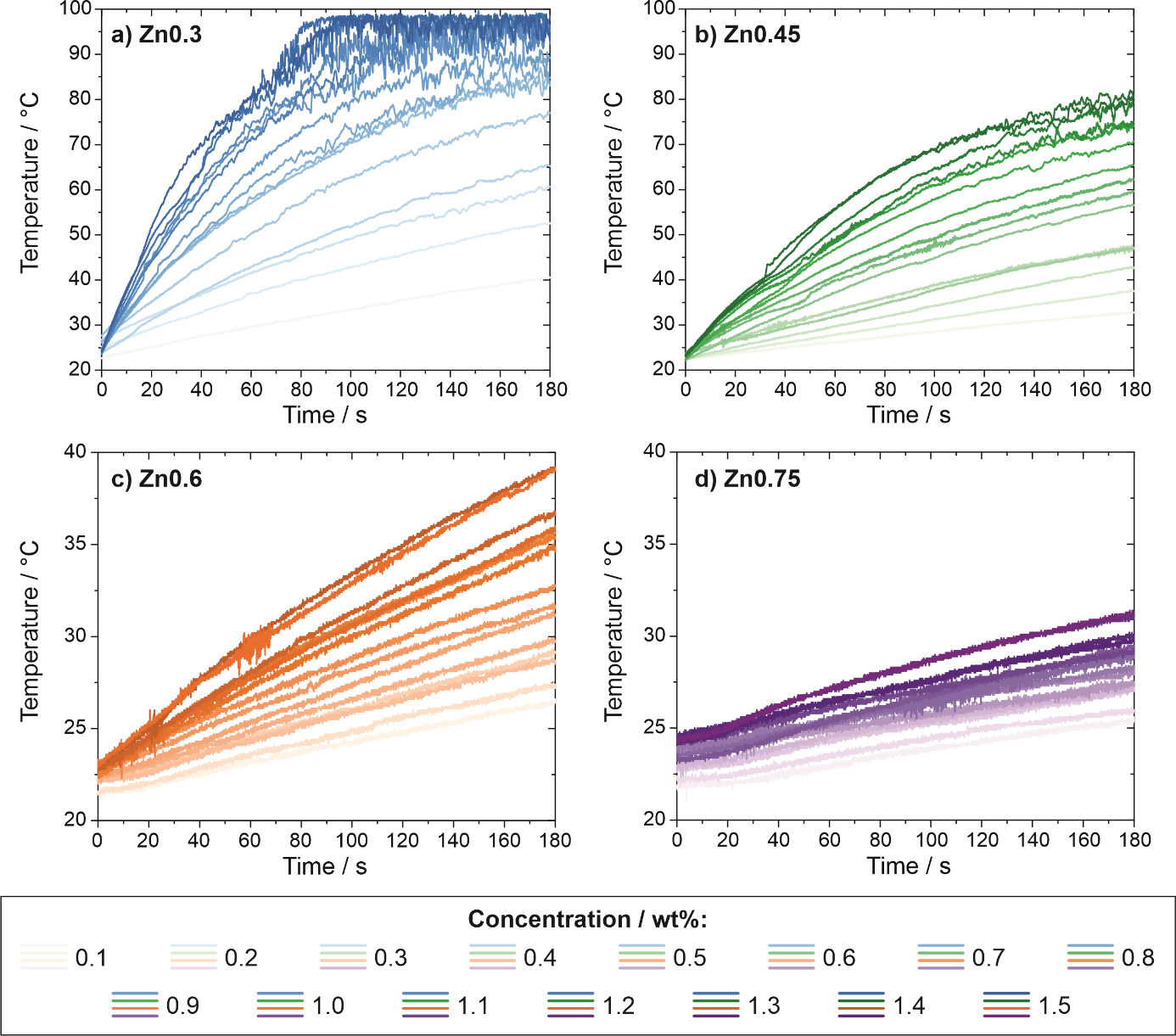


**Figure S15.** Unsmoothed induction heating curves in dispersion of Zn0.3 (a), Zn0.45 (b), Zn0.6 (c), and Zn0.75 (d) NPs, measured in different concentrations between 0.1 and 1.5 wt% at an AMF amplitude of 500 Oe and a frequency of 1.4 MHz.

**Table S10.** SAR values of Zn ferrite NPs given in W g^-1^ from concentrations of 1.0 to 1.5 wt%. SAR values are given in W per gram of the utilized nanoparticle mass.

|  | **Zn0.3 (1000 °C)** | **Zn0.3 (1100 °C)** | **Zn0.45** | **Zn0.6** | **Zn0.75** |
| --- | --- | --- | --- | --- | --- |
| **1.0 wt%** | 28.0 | 379.6 | 156.9 | 40.7 | 18.4 |
| **1.1 wt%** | 20.0 | 411.9 | 134.3 | 29.3 | 11.1 |
| **1.2 wt%** | 19.7 | 350.8 | 143.7 | 28.1 | 14.6 |
| **1.3 wt%** | 19.3 | 361.7 | 144.9 | 28.4 | 11.3 |
| **1.4 wt%** | 16.8 | 362.2 | 148.7 | 32.2 | 9.0 |
| **1.5 wt%** | 20.7 | 350.5 | 137.0 | 32.9 | 14.5 |
| **Average** | **20.8** | **369.4** | **144.2** | **31.9** | **13.1** |
| **Standard deviation** | 3.8 | 23.3 | 8.2 | 4.7 | 3.4 |


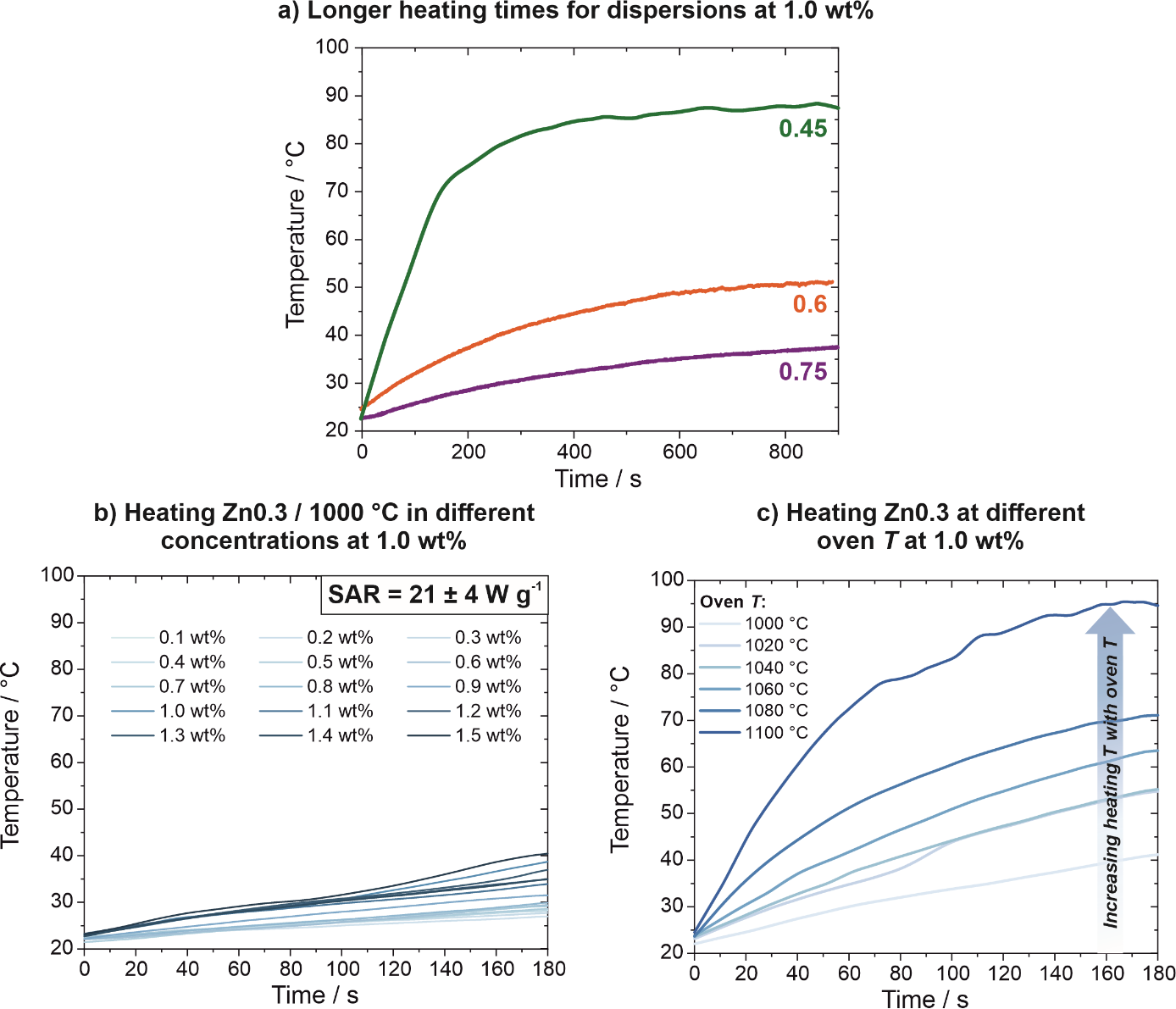


**Figure S16.** a) Induction heating curves of Zn0.45, Zn0.6, and Zn0.75 NP dispersions annealed at 1100 °C at a concentration of 1.0 wt% each, recorded over 900 s. b) Induction heating curves of Zn0.3 NPs annealed at 1000 °C in dispersion at concentrations between 0.1 and 1.5 wt%, recorded over 180 s, displaying a SAR of 21 W g^-1^. c) Induction heating curves of Zn0.3 NPs annealed at different oven temperatures at 1.0 wt%, recorded over 180 s. An increasing annealing temperature resulted in an increasing maximum reached temperature during dispersion heating. All induction heating curves were recorded at an AMF amplitude of 500 Oe and a frequency of 1.4 MHz. SAR values are given in W per gram of the utilized nanoparticle mass.

**Table S11.** Structure models used for quantitative XRD evaluation with software TOPAS, obtained from the Inorganic Crystal Structure Database (ICSD; FIZ Karlsruhe, Germany).

| **Phase** | **Formula** | **ICSD#** | **Space group** | **Authors** |
| --- | --- | --- | --- | --- |
| Magnetite | Fe_3_O_4_ | 65339 | Fd-3m | M. E. Fleet^[4]^ |
| Hematite | α-Fe_2_O_3_ | 82902 | R-3c | H. Sawada^[5]^ |
| ε-Fe_2_O_3_ | ε-Fe_2_O_3_ | 161785 | Pna2_1_ | S. Sakurai et al.^[6]^ |

**References**

[1] a) K. L. Livesey, S. Ruta, N. R. Anderson, D. Baldomir, R. W. Chantrell, D. Serantes, *Beyond the blocking model to fit nanoparticle ZFC/FC magnetisation curves*, *Scientific Reports* **2018**, *8*, 11166; b) F. Tournus, A. Tamion, *Magnetic susceptibility curves of a nanoparticle assembly II. Simulation and analysis of ZFC/FC curves in the case of a magnetic anisotropy energy distribution*, *Journal of Magnetism and Magnetic Materials* **2011**, *323*, 1118–1127; c) A. Demortière, P. Panissod, B. P. Pichon et al., *Size-dependent properties of magnetic iron oxide nanocrystals*, *Nanoscale* **2011**, *3*, 225–232.

[2] a) O. Ben Dor, S. Yochelis, I. Felner, Y. Paltiel, *Unusual ZFC and FC magnetic behavior in thin Co multi-layered structure*, *Journal of Magnetism and Magnetic Materials* **2017**, *428*, 357–361; b) K. M. Kirkpatrick, B. H. Zhou, P. C. Bunting, J. D. Rinehart, *Quantifying superparamagnetic signatures in nanoparticle magnetite: a generalized approach for physically meaningful statistics and synthesis diagnostics*, *Chemical Science* **2023**, *14*, 7589–7594.

[3] D. Serantes, M. Pereiro, R. Chantrell, D. Baldomir, *Scaling the effect of the dipolar interactions on the ZFC/FC curves of random nanoparticle assemblies*, *Journal of Magnetism and Magnetic Materials* **2018**, *460*, 28–33.

[4] M. E. Fleet, *The structure of magnetite: Symmetry of cubic spinels*, *Journal of Solid State Chemistry* **1986**, *62*, 75–82.

[5] H. Sawada, *An electron density residual study of α-ferric oxide*, *Mater. Res. Bull.* **1996**, *31*, 141–146.

[6] S. Sakurai, J. Jin, K. Hashimoto, S.-I. Ohkoshi, *Reorientation Phenomenon in a Magnetic Phase of ε-Fe_2_O_3_ Nanocrystal*, *Journal of the Physical Society of Japan* **2005**, *74*, 1946–1949.
